# Comparative Single‐Cell Transcriptomic Landscape Reveals the Regulatory Mechanisms of Lactation during Selective Breeding in Asian Water Buffalo

**DOI:** 10.1002/advs.202508847

**Published:** 2025-07-11

**Authors:** Dongmei Dai, Jingfang Si, Li Jiang, Bo Han, Kailai Wang, Xue Wang, Shihui Yan, Yichang Yin, Wei Chen, Huaming Mao, Alfredo Pauciullo, Shang‐Tong Li, Lingzhao Fang, Yi Zhang

**Affiliations:** ^1^ State Key Laboratory of Animal Biotech Breeding National Engineering Laboratory for Animal Breeding Key Laboratory of Animal Genetics, Breeding and Reproduction of Ministry of Agriculture and Rural Affairs College of Animal Science and Technology China Agricultural University Beijing 100193 China; ^2^ Glbizzia Biosciences Co., Ltd Beijing 102609 China; ^3^ Dehong Animal Husbandry Station Dehong 678400 China; ^4^ Yunnan Provincial Key Laboratory of Animal Nutrition and Feed Science Faculty of Animal Science and Technology Yunnan Agricultural University Kunming 650201 China; ^5^ Department of Agricultural, Forest and Food Sciences University of Turin Grugliasco (TO) 10095 Italy; ^6^ Center for Quantitative Genetics and Genomics (QGG) Aarhus University Aarhus 8000 Denmark

**Keywords:** comparative transcriptomics, milk production, single‐cell RNA sequencing, water buffalo

## Abstract

Characterizing the cell type‐specific transcriptome is crucial for understanding the cellular and molecular regulatory mechanisms underlying adaptive evolution and complex phenotypes. Here, single‐cell/nucleus RNA sequencing (sc/snRNA‐seq) is used to construct a cell transcriptomic atlas of 397,011 cells, representing 57 cell types, from 12 tissues in river and swamp buffalo, which exhibit significant divergence in milk production. Differential expression analyses identify metabolic and secretory tissues (i.e., liver, mammary gland, and pituitary) and cell types (e.g., hepatocytes, luminal cells, somatotropes, and lactotropes) that mediate the divergence of milk production. Lactotrope‐specific downregulation of *TRHDE* in river buffalo is associated with high milk production. Integrative analyses of sc/snRNA‐seq data with genomic data in buffalo and cattle reveal key cell types (e.g., luminal cells and excitatory neurons) and genes (e.g., *RPL13* and *LALBA*) associated with milk production. Ultimately, the Buffalo Cell Atlas (http://bovomicshub.com) will serve as a valuable resource for advancing buffalo genetics and genomics research, enabling cross‐species comparative transcriptome studies and providing deeper insights into the regulation of milk synthesis and secretion.

## Graphical abstract

In this study, a comprehensive single‐cell transcriptome atlas of river and swamp buffalo, encompassing 12 tissues and 397,011 cells, is constructed. Differential expression analyses identify metabolic and secretory tissues and cell types that mediate the divergence of milk production. Integrative analyses of sc/snRNA‐seq data with genomic data in buffalo and cattle reveal key cell types and genes associated with milk production. These results provide deeper insights into the regulation of milk synthesis and secretion.

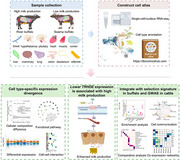

## 1 Introduction

The Asian water buffalo (*Bubalus bubalis*) is a crucial source of milk, meat, and draft power across more than 67 countries, supporting more people than any other livestock.^[^ ^1^, ^2^ ^]^ Water buffalo are broadly classified into river buffalo (*Bubalus bubalis bubalis*) and swamp buffalo (*Bubalus bubalis carabanesis*).^[^ ^3^, ^4^ ^]^ These two buffalo types diverged 0.84 million years ago^[^ ^5^ ^]^ and were independently domesticated,^[^ ^6^, ^7^, ^8^ ^]^ exhibiting distinct performance traits.^[^ ^9^ ^]^ River buffaloes are primarily used for milk production, known for the high nutritional value of their components (e.g., fat, protein, and lactose),^[^ ^10^ ^]^ whereas swamp buffaloes are primarily used for draft purposes.^[^ ^9^ ^]^ Therefore, these two buffalo types are considered excellent models for exploring the cellular and molecular regulatory mechanisms underlying milk production.

Previous studies on water buffalo haveprimarily focused on DNA variants to reveal the genomic and phenotypic diversity.^[^ ^11^, ^12^, ^13^, ^14^, ^15^, ^16^ ^]^ Although there are a limited number of tissue expression profiles for buffalo,^[^ ^17^, ^18^ ^]^ the cellular and molecular regulatory mechanisms underlying milk production traits remain unclear. The development of single‐cell omics has greatly facilitated the exploration of specific cellular and molecular mechanisms underlying complex phenotypes and adaptive evolution.^[^ ^19^, ^20^, ^21^, ^22^ ^]^ For instance, the Cattle Cell Atlas highlighted the significance of nerve cells in defining milk production traits and germline cells in determining sperm traits.^[^ ^22^ ^]^ The Human Cell Atlas will serve as a cell census, 3D map, temporal developmental map, genotype‐to‐phenotype map, and multi‐modal foundation for cell biology, providing deep insights into the mechanisms of human health and disease.^[^ ^23^ ^]^ Therefore, exploring the cellular components and gene expression networks that regulate milk production at the single‐cell level in buffalo is of significant interest.

In this study, we constructed a single‐cell transcriptomic atlas comprising 397,011 cells from 12 tissues, identifying 57 distinct cell types, in both river and swamp buffalo (two biological replicates per breed). We identified metabolic, secretory, and neuronal cell types, along with key genes and ligand‐receptor pairs associated with the divergence in milk production between these two buffalo types. Additionally, we identified conserved genetic and cellular mechanisms of lactation across buffalo, cattle, and humans at the single‐cell level. In summary, our Buffalo Cell Atlas (http://bovomicshub.com) provides an invaluable resource for characterizing the transcriptomic and genomic features of buffalo and offers novel insights into the cellular mechanisms of milk production.

## 2 Results

### 2.1 Construction of a Single‐Cell Atlas in Two Buffalo Types

**Figure 1:**
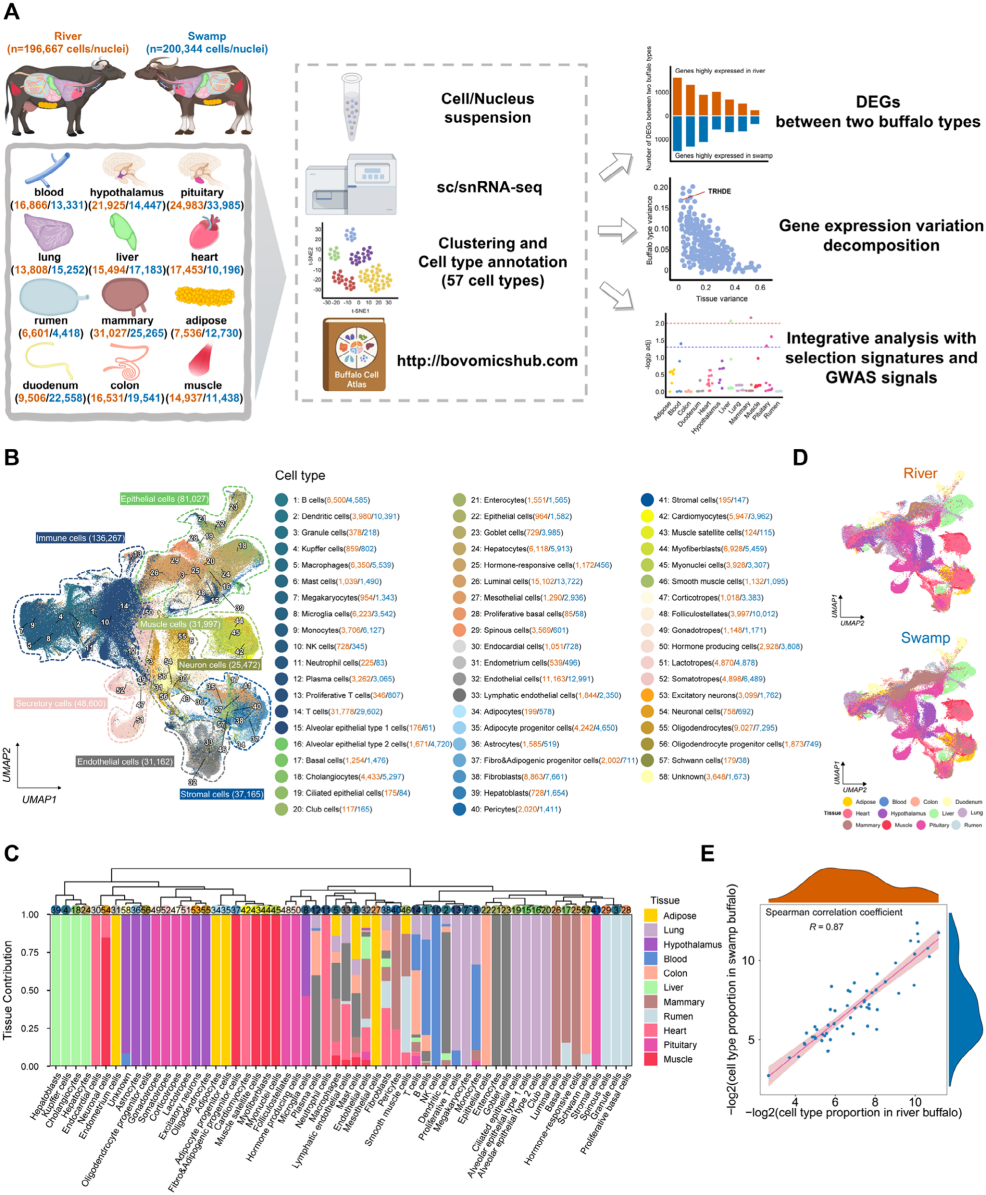
Construction of a cell atlas across 12 tissues of the river and swamp buffalo. A) A schematic diagram of 12 buffalo tissues collected in this study and the overview of sc/snRNA‐seq data analysis workflow. The number of cells/nuclei in each tissue is shown in the river and swamp buffalo, respectively. The schematic diagram is created by Fig draw. B) UMAP visualization of all clusters colored by major cell types. A total of 57 cell types from seven major cell lineages are identified in the dataset. Cell type annotation and number of cells in each cell type are provided in the legend to the right. The orange numbers in parentheses indicate the number of cells for river buffalo, and the blue numbers indicate those for swamp buffalo. C) The hierarchical clustering of cell type‐specific transcriptome and the relative contributions of tissues to each cell type. D) UMAP visualization of global clustering of all cells from the dataset, colored by tissues. E) The correlation of cellular composition between river and swamp buffalo. Each dot represents the cellular proportion in the two buffalo types.

To explore the molecular and cellular mechanisms underlying phenotypic divergence between river and swamp buffalo, we collected 12 tissues from four early lactation buffaloes belonging to Binglangjiang (river type) and Dehong (swamp type) breeds (Table S1, Supporting Information), including mammary gland, pituitary gland, rumen, duodenum, colon, lung, blood, hypothalamus, heart, liver, adipose tissue, and muscle. In total, we generated single‐cell/nucleus RNA sequencing data for 48 samples (**Figure** **1**A; Table S2, Supporting Information). After stringent quality control and batch correction (Figures S1 and S2, and Table S2, Supporting Information), we found that neither individual‐sample nor batch variation affected the global data structure, suggesting that the correction was adequate. A total of 397,011 cells/nuclei (hereinafter referred to as cells) were retained for further analysis, with 196,667 and 200,344 from the river and swamp types, respectively (Table S3, Supporting Information). The number of cells ranges from 11,019 in the rumen to 58,968 in the pituitary, with a median of 1,187 genes and 2,046 unique molecular identifiers (UMIs) per cell (Figure S3 and Table S4, Supporting Information). Our Buffalo Cell Atlas can be searched interactively by tissue, cell type, and gene (Figures S4 and S5, Supporting Information) through the website (http://bovomicshub.com).

To explore cellular heterogeneity across tissues and buffalo types, we generated visualizations using Uniform Manifold Approximation and Projection (UMAP) plots and annotated the cell clusters based on canonical marker genes (Tables S5 and S6, Supporting Information). In total, we identified 57 major cell types across the 12 tissues, with an average of 10 cell types in each tissue (Figure 1B; Figures S6 and S7, Supporting Information). Out of the 57 major cell types, 39 appear in one tissue only, indicative of their tissue‐specific functions, such as lactotropes in the pituitary gland, and luminal cells in the mammary gland (Figure 1C). Additionally, certain cell types exhibited widespread expression patterns across multiple tissues; for instance, macrophages were present in adipose tissue, colon, duodenum, heart, lung, mammary gland, and muscle (Figure 1C). UMAP visualization of cells from the two biological replicates revealed highly consistent cell composition and gene expression patterns, demonstrating high reproducibility (Figures S8 and S9, Supporting Information). The global cell clustering patterns were highly similar between the two buffalo types (Figure 1D; Figure S10A,B, Supporting Information), with a strong correlation in cellular composition (*R* = 0.87, Figure 1E; Figure S10C, Supporting Information), suggesting that the overall cellular composition is largely consistent between these two buffalo types.

### 2.2 Differential Analyses of Cell Types and Genes Between River and Swamp Buffalo

**Figure 2:**
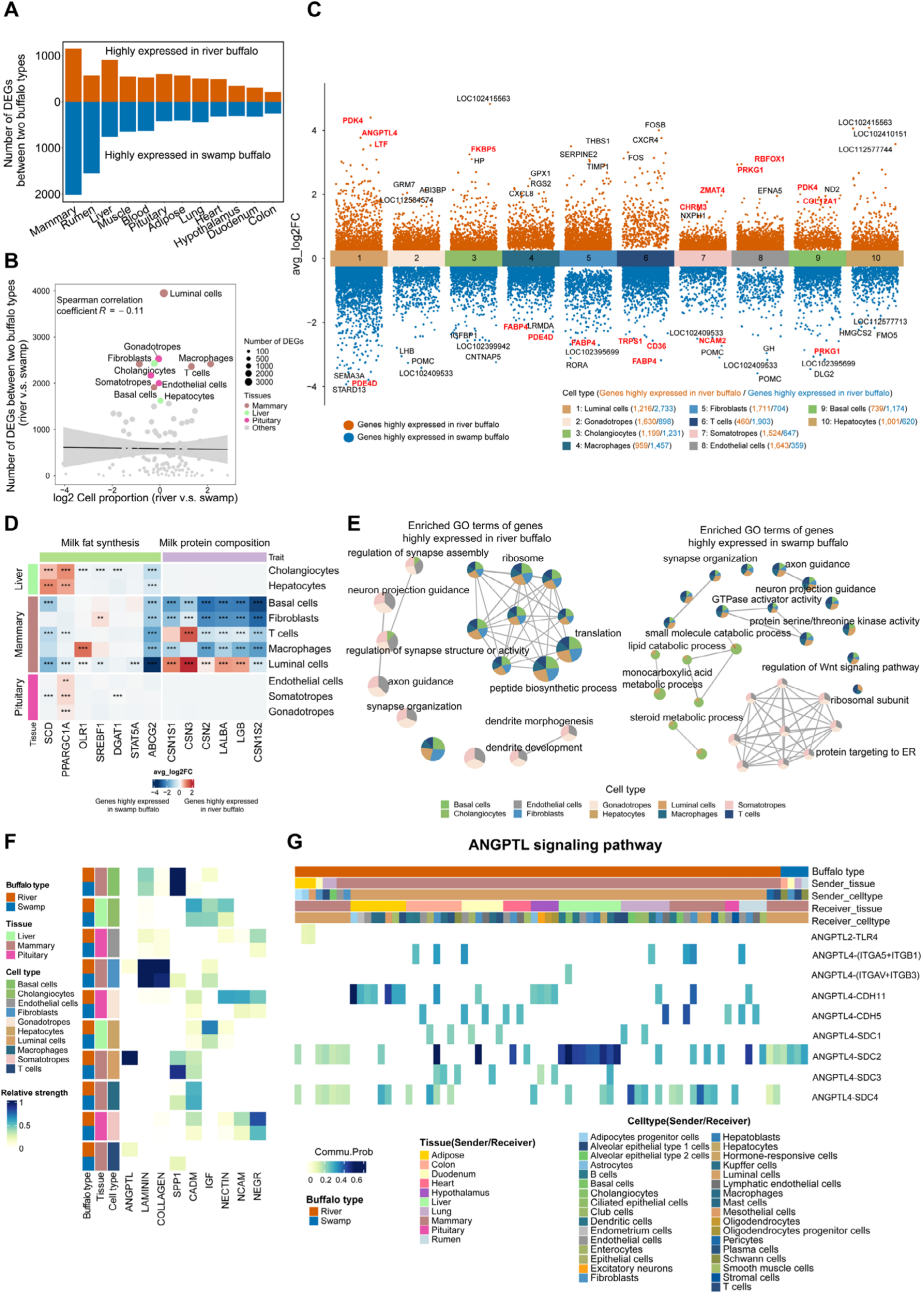
Cell type‐specific gene expression changes between river and swamp buffalo. A) The bar plot showing the number of differentially expressed genes (DEGs) between river and swamp buffalo across tissues. Orange represents genes highly expressed in river buffalo, and blue represents genes highly expressed in swamp buffalo. B) Spearman's correlation of the number of DEGs and relative cellular composition differences between the two buffalo types. The top 10 cell types with the highest number of DEGs and the corresponding tissues are highlighted. C) The expression changes of genes in the top 10 cell types. The genes highlighted in red and bold font represent those previously reported to be associated with milk production. D) Heat map showing the expression differences of key genes associated with milk fat synthesis and protein composition in river and swamp buffalo across the top 10 cell types highlighted in (B) and (C). Statistical significance is denoted as follows: ^*^ denotes p_adj < 0.05, ^**^ denotes p_adj < 0.01, ^***^ denotes p_adj < 0.001. E) GO enrichment analysis of DEGs in the top 10 cell types highlighted in (B) and (C). F) Heatmap showing the relative strength of cell–cell interactions between the top 10 cell types and other cell types within lactation‐related signaling pathways in river and swamp buffalo. G) Heatmap representing the communication probability of key cell‐cell interactions in the ANGPTL signaling pathway in river and swamp buffaloes.

To further investigate the alteration in the transcription profiles between the two buffalo types, we performed differential expression analysis at both the tissue and cell type levels (**Figure** **2**A,B). Notably, the mammary gland, rumen, and liver exhibited the highest number of differentially expressed genes (DEGs) between the two buffalo types (Figure 2A), reflecting their underlying divergence in milk production resulting from selective breeding. The lack of correlation between the number of DEGs and cell numbers (Figure 2B) suggested that the number of DEGs can reliably identify critical cell types contributing to phenotypic variation. The top 10 cell types with the highest number of DEGs included luminal cells, gonadotropes, cholangiocytes, macrophages, fibroblasts, T cells, somatotropes, endothelial cells, basal cells, and hepatocytes, primarily found in the mammary gland, pituitary, and liver (Figure 2B; Figure S11, Supporting Information).

Within these DEGs, we identified key genes influencing milk production traits, such as *ANGPTL4* ^[^ ^24^ ^]^ and *FKBP5* ^[^ ^25^ ^]^ (Figure 2C). Furthermore, among the previously reported key genes associated with milk proteins (*CSN1S1*, *CSN1S2*, *CSN3*, *CSN2*, *LALBA*, and *LGB*)^[^ ^12^, ^26^, ^27^, ^28^, ^29^, ^30^ ^]^ and milk fat metabolism (*SCD*, *PPARGC1A*, *OLR1*, *SREBF1*, *DGAT1*, *STAT5A*, and *ABCG2*),^[^ ^31^, ^32^, ^33^, ^34^, ^35^, ^36^, ^37^, ^38^, ^39^, ^40^, ^41^, ^42^ ^]^ most milk protein genes (i.e., *CSN1S1*, *CSN3*, *LALBA*, and *LGB*) were highly expressed in the luminal cells of river buffalo rather than swamp buffalo (Figure 2D). *SCD* and *PPARGC1A* exhibited higher expression in the cholangiocytes and hepatocytes of river buffalo compared to those of swamp buffalo, while *ABCG2* was highly expressed in the luminal cells of swamp buffalo, but not in those of river buffalo (Figure 2D). These lactation‐relevant genes mentioned above were generally upregulated in river buffalo compared to swamp buffalo, indicating their important roles in milk production. Gene ontology (GO) analysis revealed that genes upregulated in river buffalo were predominantly associated with protein metabolism regulation and neurobiology, including translation, peptide biosynthetic processes, and neuron projection guidance (Figure 2E). In contrast, GO terms associated with upregulated genes in swamp buffalo were largely related to metabolic processes and the regulation of enzyme activity (Figure 2E).

In lactation‐related signaling pathways, we also identified noticeable differences in cell–cell interactions between the top 10 cell types and other cell types in river and swamp buffalo (Figure 2F). For instance, in the ANGPTL signaling pathway, luminal cells in river buffalo exhibited stronger communication with other cell types than those in swamp buffalo (Figure 2F), and luminal cells were identified as the major sender (Figure 2G). Notably, we found that the ANGPTL4–SDC2 pair, previously associated with milk production traits,^[^ ^43^, ^44^ ^]^ exhibited the highest specificity in interactions between luminal cells and liver cell types in river buffaloes (Figure 2G). These findings further indicated the critical roles of these divergent cell types and genes in lactation.

### 2.3 Lactotrope‐Specific Downregulation of *TRHDE* is Associated with High Milk Production

**Figure 3:**
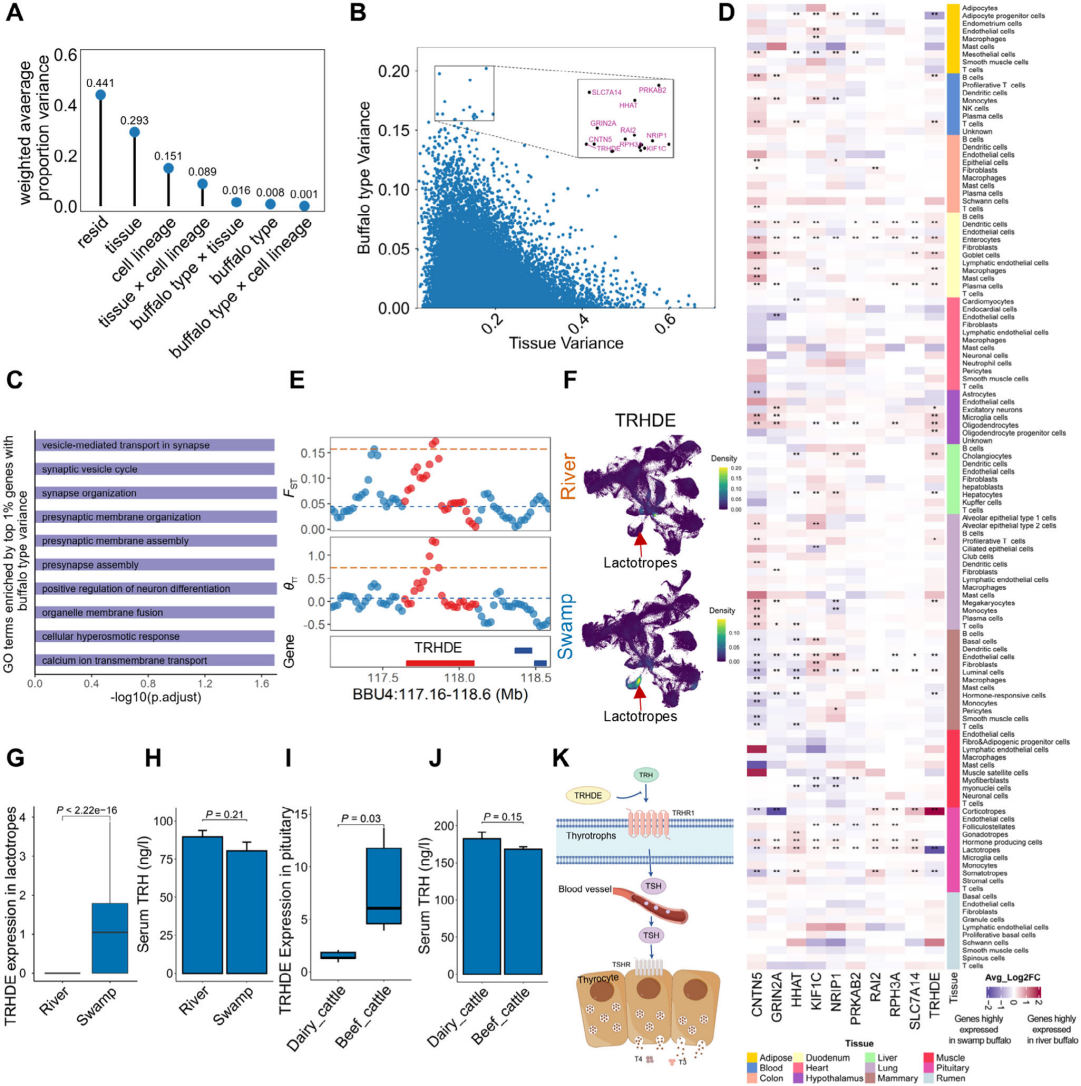
Lactotrope‐specific downregulation of *TRHDE* in river buffalo is associated with high milk production. A) Principal variance component analysis showing the gene expression variance contributed by factors such as buffalo type, tissue, cell lineage, and their interactions. B) Gene‐level variance decomposition of contribution by buffalo types and tissues to gene expression variation. C) GO enrichment analysis of the top 1% genes with buffalo type variance. D) Heatmap showing the expression differences of the top 10 genes with high buffalo type variance in all cell types of the two buffalo types. Statistical significance is denoted as follows: ^*^ denotes p_adj < 0.05, ^**^ denotes p_adj < 0.01. E) Selection signatures, indicated by *F*_ST_ and 𝜃*π* values, in the genomic region around *THRDE* in dairy river buffalo, based on whole‐genome sequencing of buffalo populations. F) Kernel density visualization of *TRHDE* expression in river and swamp buffalo using Nebulosa, with lactotropes indicated by the arrow. G) *TRHDE* expression in lactotropes of river and swamp buffalo. Statistical significance is tested using a two‐tailed *t*‐test. H) The concentration of TRH in the serum of river and swamp buffalo. (*n* = three biological replicates; mean ± SEM). Statistical significance is tested using a two‐tailed *t*‐test. I) *TRHDE* expression in the pituitary of dairy and beef cattle. (*n* = five biological replicates). Statistical significance is tested using a two‐tailed *t*‐test. J) The concentration of TRH in the serum of dairy and beef cattle. (*n* = three biological replicates; mean ± SEM). Statistical significance is tested using a two‐tailed *t*‐test. K) A schematic diagram illustrating the function of *TRHDE* in regulating TRH signaling which was created by Fig draw. TRH: thyrotropin releasing hormone; TSH: thyroid‐stimulating hormone; T3 and T4: thyroid hormones.

To evaluate the contribution of various factors such as buffalo type, tissue, cell lineage, and their interactions to gene expression variance, we performed principal variance component analysis (PVCA). Our result revealed that the tissue accounted for the largest proportion of the global variance, explaining 29.3% of the variation in gene expression (**Figure** **3**A). Notably, the interaction between buffalo type and tissue (1.6%) explained a larger proportion of variance than buffalo type alone (0.8%) (Figure 3A), indicating a tissue‐specific effect of buffalo type on gene expression profiles. The top 1% of genes with highest variance explained by buffalo type, identified by a linear mixed model (Figure 3B), were primarily involved in neuronal activity, such as the positive regulation of neuron differentiation, thus indicating a pivotal role of neural regulation in the divergence between the two buffalo types (Figure 3C). Among the top 10 genes with the highest variance explained by buffalo type, including *PRKAB2*, *SLC7A14*, *HHAT*, *GRIN2A*, *RAI2*, *RPH3A*, *NRIP1*, *CNTN5*, *TRHDE*, and *KIF1C* (Figure 3B), *TRHDE* showed significant expression differences between the two buffalo types in key milk‐related cell types, including lactotropes, somatotropes, cholangiocytes, and adipocyte progenitor cells (Figure 3D). *TRHDE* encodes a thyrotropin releasing hormone degrading ectoenzyme that specifically cleaves and inactivates thyrotropin‐releasing hormone (TRH),^[^ ^45^ ^]^ and it was under strong selection in dairy river buffalo (Figure 3E).^[^ ^12^ ^]^ *TRHDE* was lowly expressed in lactotropes of river buffalo (Figure 3F,G; Figure S12A, Supporting Information); consequently, serum TRH levels were higher in river buffalo than in swamp buffalo (Figure 3H). Furthermore, this downregulation pattern of *TRHDE* expression was conserved between cattle and river buffalo (Figure 3I,J). TRH has been reported to stimulate the secretion of thyroid‐stimulating hormone (TSH), growth hormone (GH), and prolactin (PRL), and is associated with milk performance in dairy cattle.^[^ ^46^, ^47^, ^48^ ^]^ Our results indicated that lower *TRHDE* expression in lactotropes was associated with increased TRH levels, which in turn may promote thyroid hormone release (Figure 3K). We further analyzed the expression of the well‐known lactation‐related genes *PRL* and *GH*, and found that they were downregulated in pituitary cell types of river buffalo, whereas their receptor genes, *PRLR* and *GHR*, were highly expressed in hormone‐responsive cells of river buffalo (Figure S12B, Supporting Information). Taken together, lower *TRHDE* expression in lactotropes, along with high expression of *PRLR* and *GHR* in hormone‐responsive cells, may collectively contribute to enhanced milk yield in river buffalo.

### 2.4 Cell Types and Intercellular Communications under Selection in River Buffalo

**Figure 4:**
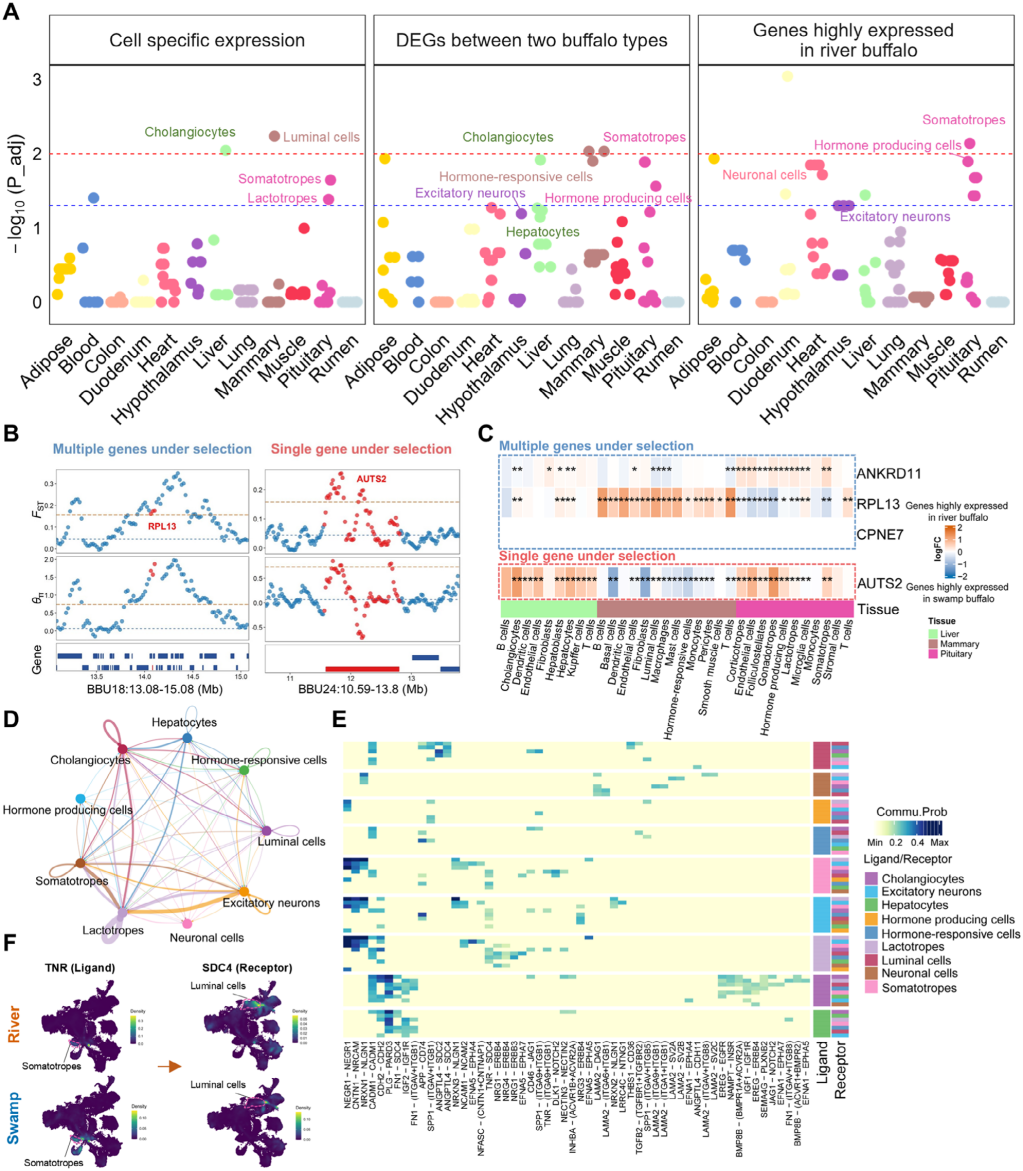
Cell types and intercellular communication under selection in river buffalo. A) Manhattan plots showing the associations between regions under selection and buffalo cell types, calculated using three gene sets in the top panel. Each dot represents an association between the cell type and regions under selection. The red and blue lines represent the highly significant and significant levels for each cell type, corresponding to *P*‐values of 0.01 and 0.05, respectively. B) Selection signatures, indicated by *F*_ST_ and 𝜃*π* values, in the genomic region around *RPL13* and *AUTS2* in dairy river buffalo, based on whole‐genome sequencing data of buffalo populations. C) Heatmap showing expression differences of the same genes as in (B) in liver, mammary, and pituitary cell types. Statistical significance is denoted as follows: ^*^ denotes p_adj < 0.05, ^**^ denotes p_adj < 0.01. D) Cellular communication analysis between nine crucial cell types associated with milk production traits, where the width of intercellular connections represents the strength of communication. E) Key ligand‐receptor pairs associated with milk production traits, identified in core cell types associated with milk production, as shown in (D). The darkness of the color shows the communication probability. F) Kernel density visualization of the expression of a ligand‐receptor pair secreted by somatotropes and targeting luminal cells using Nebulosa.

To investigate whether cell types and intercellular communications were under selection in river buffalo, we analyzed 122 genes identified as under selection in river buffalo (Table S7, Supporting Information)^[^ ^12^ ^]^ and performed enrichment analysis for those genes across three gene sets, including cell‐type‐specific genes, DEGs between the two buffalo types, and genes highly expressed in river buffalo (**Figure** **4**A). Among the 20 cell types showing significant or near‐significant enrichment (Table S8, Supporting Information), nine were involved in regulating milk production,^[^ ^22^, ^49^, ^50^, ^51^, ^52^, ^53^, ^54^ ^]^ including luminal cells, cholangiocytes, hepatocytes, lactotropes, somatotropes, hormone‐responsive cells, hormone producing cells, excitatory neurons, and neuronal cells (Figure 4A). This finding supports the crucial role of gene transcription modulation in neuronal, secretory, and metabolic cell types in shaping milk production phenotypes during artificial selection. Among the three gene sets across nine cell types, 41 genes were found to be under selection in river buffalo (Table S9, Supporting Information), providing a cellular perspective for prioritizing genes under selection directly associated with milk production. For instance, in a genomic region under selection containing multiple genes, *RPL13* was inferred to be a potential key gene associated with milk production, as it was the only gene showing high expression in the luminal cells of river buffalo rather than swamp buffalo (Figure 4B,C). In another genomic region under selection containing a single gene, *AUTS2* was highly expressed in the pituitary and liver cell types of river buffalo (Figure 4B,C), which has been reported to be associated with milk fat and protein traits,^[^ ^55^ ^]^ further supporting its association with milk production.

We further explored potential cell interactions across the nine cell types identified in the above analysis (Figure 4D). Significant interactions were observed among lactotropes, somatotropes, cholangiocytes, excitatory neurons, luminal cells, and hepatocytes (Figure 4D), suggesting co‐regulations of these cell types in milk production during long‐term artificial selection. Several ligand‐receptor pairs were implicated in milk production traits, including the TNR‐(ITGA9+ITGB1) pair, which was involved in neuronal and mammary gland development, as well as milk composition regulation^[^ ^56^, ^57^, ^58^, ^59^ ^]^ (Figure 4E). We identified the receptor *SDC4* as a potential key gene associated with milk production traits, based on the ligand‐receptor relationship with ligand *TNR* and its expression in luminal cells (Figure 4F). SDC4 plays a crucial role in the function of major metabolic tissues and serves as a regulator of lipid metabolism,^[^ ^60^, ^61^, ^62^ ^]^ which further supports its potential involvement in lactation.

### 2.5 Cross‐Species Similarities in Molecular and Cellular Mechanisms of Milk Production Traits

**Figure 5:**
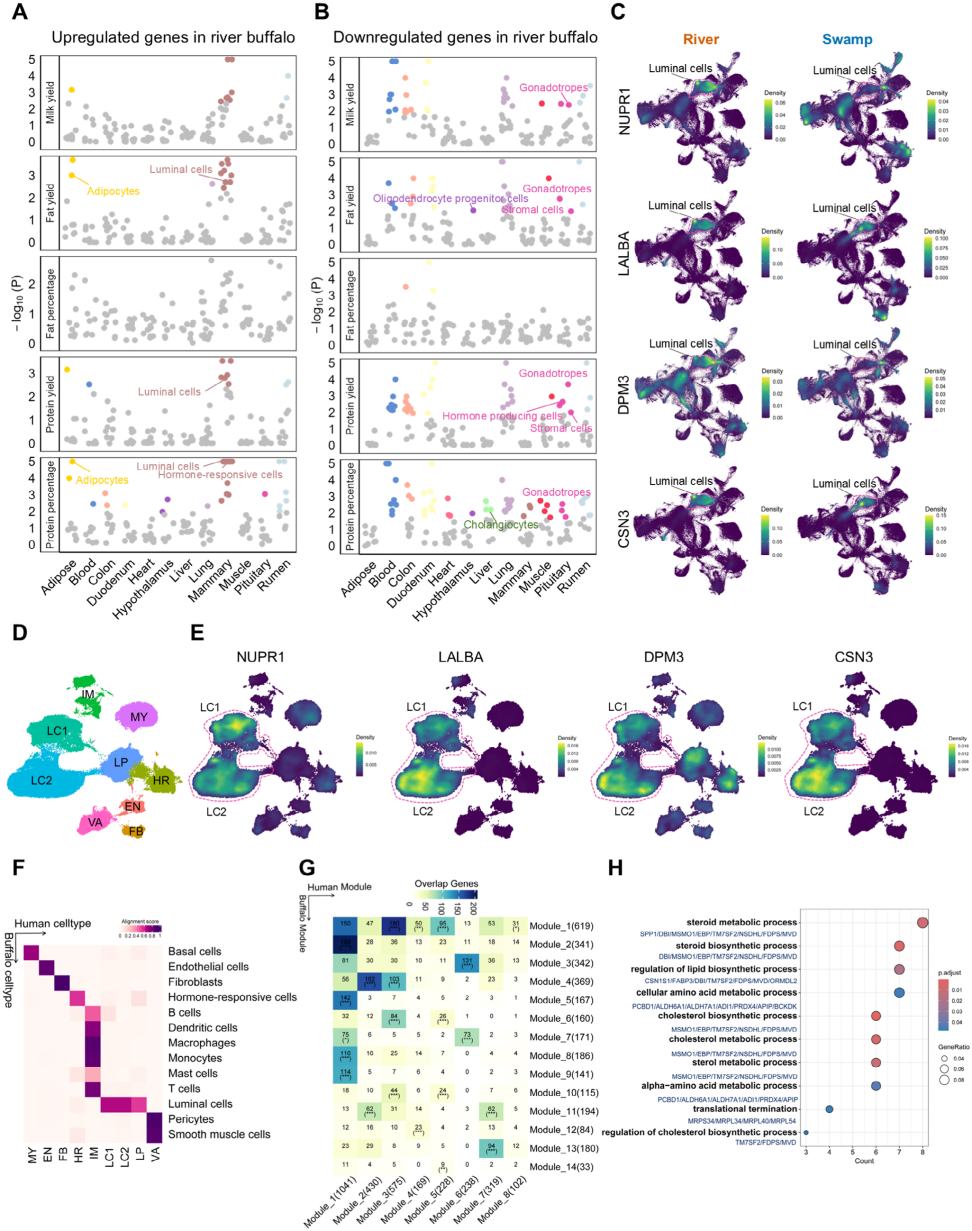
Cross‐species similarities in cellular and molecular mechanisms underlying lactation. A, B) Manhattan plots showing the associations between milk production traits and cell types, computed using a sum‐based GWAS signal enrichment analysis for the top 700 upregulated (A) and downregulated (B) genes in river buffalo with a 20‐kb extension. Each dot represents a cell‐type‐trait association. Dots with adjusted‐*P* (FDR) < 0.05 are colored according to the corresponding tissue colors in Figure 4A. C) Kernel density visualization of lactation‐related gene expression between the two buffalo types using Nebulosa. D) UMAP dimensional reduction of the mammary cells in human reveals distinct clusters arising from human mammary tissue and milk. A total of nine cell types are identified in the dataset from humans. MY, myoepithelial; HR, luminal hormone‐responsive; LP, luminal progenitor; LC1, luminal cluster 1; LC2, luminal cluster 2; IM, immune; VA, vascular accessory; EN, endothelial; FB, fibroblasts. E) Kernel density visualization of lactation‐related gene expression in human mammary tissue and milk using Nebulosa. F) Heatmap showing alignment scores that indicate transcriptome similarity between buffalo mammary and human mammary cell types. G) Correspondence of human and buffalo modules. Statistical significance is denoted as follows: ^*^ denotes p_adj < 0.05, ^**^ denotes p_adj < 0.01, ^***^ denotes p_adj < 0.001. H) GO enrichment analysis of genes shared between buffalo module 13 and human module 7.

Buffalo shared convergent signatures and gene expression patterns with cattle during selective breeding^[^ ^16^, ^17^ ^]^ The regularly recorded milk production traits in cattle include milk yield, milk fat yield, milk protein yield, milk fat percentage, and milk protein percentage.^[^ ^63^ ^]^ To gain further insights into the relevance of cell types to milk production traits, we downloaded genome‐wide association study (GWAS) summary statistics for these five milk production traits in cattle and performed enrichment analyses for up‐ and down‐regulated genes in river buffalo versus swamp buffalo (**Figure** **5**A,B). We identified 20 cell types significantly associated with five milk production traits (adjusted *P* < 0.05, Table S10, Supporting Information) through enrichment analyses of upregulated genes in river buffalo. Notably, luminal cells, hormone‐responsive cells, and adipocytes showed significant associations with milk production traits (Figure 5A; Table S10, Supporting Information). In contrast, enrichment analyses of downregulated genes revealed a greater diversity of cell types, such as stromal cells, gonadotropes, and hormone‐producing cells (adjusted *P* < 0.05, Figure 5B; Table S11, Supporting Information). These findings support the crucial roles of these cell types in the regulation of milk production. For instance, genes related to milk fat and protein, such as *LALBA*,^[^ ^64^ ^]^ *NUPR1*,^[^ ^65^ ^]^ *DPM3*,^[^ ^66^, ^67^ ^]^ and *CSN3* ^[^ ^26^ ^]^ were highly expressed in luminal cells of river buffalo (Figure 5C). Additionally, *LALBA*, *NUPR1*, *DPM3*, and *CSN3* were also highly expressed in luminal clusters (LC1 and LC2) of human milk (Figure 5D,E) based on the scRNA‐seq data from human.^[^ ^68^ ^]^

To further explore the similarities in cell types of the mammary gland between humans and buffalo, we used the self‐assembling manifold mapping (SAMap) algorithm to align and directly compare their molecular signatures. These cell types exhibited high inter‐species alignment scores, demonstrating conserved cellular correspondence between the species (Figure 5F). We further constructed weighted gene co‐expression networks in buffalo and humans and identified modules significantly associated with luminal cells (modules 1 and 13) and hormone‐responsive cells (modules 4, 12, and 13) in buffalo (Figure S13A,B, Supporting Information), as well as luminal clusters (modules 1, 7, and 8) in humans (Figure S13C,D, Supporting Information). Buffalo module 13 showed significant overlap with human module 7 (Figure 5G), and these genes were enriched in pathways related to protein and lipid metabolism (Figure 5H). These findings suggested a similar cellular mechanism in lactation across mammalian species.

## 3 Discussion

In this study, we generated single‐cell transcriptomic data from 397,011 cells across 12 tissues involved in the regulation of lactation in river and swamp buffalo. We created the first comparative cell atlas of these two buffalo types and developed a web portal (http://bovomicshub.com) to make the results freely and easily accessible to the research community. Our analysis revealed cellular heterogeneity across the buffalo body and identified cell type‐specific gene expression differences related to milk production divergence resulting from long‐term artificial selection.

Milk production, a key trait in buffalo selective breeding, is regulated by the coordinated actions of reproductive and metabolic hormones.^[^ ^9^, ^69^ ^]^ In this study, lactotrope‐specific downregulation of *TRHDE* was associated with increased TRH levels, which might promote milk production—a regulatory mechanism conserved in both cattle and buffalo. It was reported that TRH stimulated the secretion of TSH, GH, and PRL, and was associated with milk performance in dairy cows.^[^ ^46^, ^47^, ^48^ ^]^ TSH influences the synthesis of thyroid hormones, which regulate transcription in mammary cells and mammary gland development, thereby affecting milk production.^[^ ^70^, ^71^, ^72^, ^73^ ^]^ We found that *PRL* and *GH* were downregulated in pituitary cell types of river buffalo, whereas their receptor genes were highly expressed in hormone‐responsive cells of river buffalo. PRLR‐deficient mice showed normal side branching and the formation of alveolar buds, but no lobuloalveolar development.^[^ ^74^ ^]^ In humans, besides binding to GHR, GH can also interact with PRLR to regulate lactation, whereas PRL primarily signals through PRLR.^[^ ^75^, ^76^ ^]^ These findings suggest that PRLR plays a more critical role in lactation. The high expression of *PRLR* and *GHR* in hormone‐responsive cells of river buffalo may regulate lactation by modulating downstream signaling pathway genes (e.g., *LALBA*, *CSN2*).

Lactation depends on the secretion of numerous hormones, many of which are regulated by the hypothalamic‐pituitary axis. Functional enrichment of genes identified by DEG and PVCA analyses highlighted significant divergence in neural regulatory pathways between the two buffalo types, suggesting potential differences in neurophysiological processes in lactation. This finding aligns with previous bulk tissue‐level observations in cattle, where neurobiology was strongly associated with milk production traits.^[^ ^77^ ^]^ Of interest, we found that excitatory neurons exhibited strong communication with both lactotropes and somatotropes in these lactating buffaloes. The main ligand–receptor pairs identified between excitatory neurons and lactotropes have previously been implicated in milk production traits or mammary gland development. For instance, *NRXN1* has been reported to be associated with milk production and milk fatty acid traits,^[^ ^78^, ^79^, ^80^ ^]^ and *NLGN1* is involved in mammalian nerve development.^[^ ^81^ ^]^ In another ligand‐receptor pair, *NEGR1* is involved in intracellular cholesterol trafficking.^[^ ^82^ ^]^ Notably, a recent study also reported an association between excitatory neurons and milk fat yield in cattle.^[^ ^22^ ^]^ Therefore, it is reasonable to infer that excitatory neurons may regulate lactation by enhancing cell‐cell communication with key lactation‐related cell types.

The Buffalo Cell Atlas offers valuable insights into the cellular and molecular mechanisms underlying milk production traits. However, it still has several limitations. First, the number of individuals and breeds in this study was limited. Future work should incorporate population‐level integrative analyses of single‐cell transcriptome and whole genome sequencing data to elucidate how genetic variations influence cell type‐specific gene expression. Additionally, the function of key genes identified in this study requires further validation with a larger sample size. Second, the current atlas was static and focused solely on adult lactating buffaloes. Expanding single‐cell transcriptomic profiling across development stages and diverse environmental conditions would provide a more comprehensive understanding of the molecular drivers of milk production in buffalo. Lastly, we employed a standard and unified quality control and analysis pipeline across all tissues in this study. To further empower the research community, we plan to develop additional analytical tools within our web portal, allowing for the reanalysis of raw data from different tissues with optimized parameters.

## 4 Conclusion

Overall, this study presents a high‐resolution body‐wide single‐cell transcriptomic landscape of buffalo and highlights cell type‐specific gene expression differences associated with the divergence in milk production between river and swamp buffalo. Our scRNA‐seq analysis offers novel insights into the molecular regulation of milk synthesis and secretion and reveals the potential for cross‐species comparison, providing new perspectives on the evolutionary dynamics of cellular function in dairy species. These findings can further serve as biological priors for prioritizing causal genes and contribute to the future development of genomic selection in the species.

## 5 Experimental Section

### Sample Collection

The Binglangjiang buffalo and the Dehong buffalo are two well‐known indigenous Chinese breeds, belonging to the river and swamp buffalo, respectively. Four female first‐lactation buffaloes (two Binglangjiang buffaloes and two Dehong buffaloes), two months postpartum, comparable in age, feeding system, feed ration, and free from clinical mastitis, were obtained from the same commercial farm in Yunnan, China (Table S1, Supporting Information). Peripheral blood was drawn from the jugular vein and stored at 4 °C. Eleven tissues, including mammary, hypothalamus, pituitary, heart, liver, lung, rumen, adipose tissue, colon, duodenum, and muscle, were freshly harvested from postmortem samples. Each tissue, except for peripheral blood, was cut into 5–10 pieces of roughly 100 mg each on ice with sterilized scissors. Samples from the hypothalamus, adipose tissue, liver, heart, and muscle were transferred to cryogenic vials, snap frozen in liquid nitrogen, and stored until nuclear extraction for snRNA‐seq. Additionally, samples from the mammary, pituitary, lung, rumen, colon, and duodenum were preserved in MACS Tissue Storage Solution (Miltenyi Biotech, 130‐100‐008) at 4 °C and protected from light until dissociation for scRNA‐seq.

### Preparation of Single‐Cell Suspension

The sample (∼0.5 cm^3^) was removed from the preservation solution and washed 3 times in PBS, cut into ∼1 mm^3^ pieces with scissors, transferred into a 15 mL centrifuge tube containing multiple enzymes diluted in HBSS (Gibco, 14175095), and incubated at 37 °C for 30–60 min (Table S12, Supporting Information). The digestion was stopped by adding 10% FBS, followed by a filtration step through a 40 µm Cell Strainer. Dissociated cells were centrifuged at 500 rpm for 5 min at 4 °C, and the supernatant was discarded. Then the pellet was resuspended in 1× PBS with 0.04% BSA. The cell viability was assessed using the acridine orange/propidium iodide (AO/PI) Double Staining Kit (APExBIO, K2238).

### Preparation of Single‐Nucleus Suspension

Tissue samples (∼0.5 cm^3^) were cut into ∼2 mm^3^ pieces and homogenized using the Dounce homogenizer with 25 strokes of the loose pestle A, followed by 25 strokes of the tight pestle B in 1 mL of ice‐cold homogenization buffer supplemented with protease (Roche, CO‐RO) and RNase (Thermo Fisher Scientific, AM2696 and 10777019) inhibitor. The homogenate was filtered through a 40 µm cell strainer into a 5 mL Eppendorf pre‐chilled tube, washing the Dounce homogenizer with an additional 500 µL of cold homogenization buffer. To collect dissociated single nuclei, the sample was centrifuged at 500 g for 5 min at 4 °C, and the supernatant was discarded. After centrifugation, the nuclear pellet was resuspended using an appropriate amount of 1× PBS/0.5% BSA with RNase inhibitor (Thermo Fisher Scientific, AM2696 and 10777019), and counted using AO/PI. A final concentration of 1,000 nuclei per µL was used for capture and library generation.

### ScRNA‐Seq Library Preparation and Sequencing

The DNBelab C Series Single‐Cell Library Prep Set (MGI) or 10X Chromium system was utilized for single‐cell RNA‐seq library preparation, including droplet encapsulation, emulsion breakage, mRNA capture bead collection, reverse transcription, cDNA amplification, and purification. Indexed libraries were constructed according to the manufacturer's protocol. The sequencing libraries were quantified by Qubit ssDNA Assay Kit (Thermo Fisher Scientific, Q10212). The sequencing libraries were sequenced by the DNBSEQ T7. The buffalo genomic UOA_WB_1^[^ ^83^ ^]^ reference assembly in FASTA format and annotated gene model in GTF format were downloaded from the NCBI (https://www.ncbi.nlm.nih.gov/datasets/genome/GCF_003121395.1/). Raw scRNA‐seq data were aligned to the buffalo reference genome and subjected to barcode assignment and UMI counting using the commands recommended by DNBC4tools or Cell Ranger software.

### Single‐Cell RNA‐Seq Data Processing

Downstream analyses of scRNA‐seq data were processed with Seurat v4.3.0.^[^ ^84^ ^]^ To ensure the accuracy and robustness of the results, ambient RNA was removed using SoupX v1.6.2^[^ ^85^ ^]^ with default settings. Then, genes expressed in fewer than three cells were excluded, and cells expressing fewer than 200 genes or more than 8000 genes were removed. Additionally, cells with mitochondrial gene percent exceeding 10% were filtered out. Doublets in the data set were removed with DoubletFinder v2.0.3.^[^ ^86^ ^]^ After filtering, the function of “LogNormalize” was applied to perform data normalization, and the scale factor was set at 10,000. Subsequently, variable genes detecting and data scaling were respectively processed using functions of “FindVariableFeatures” (“vst” method, 2000 features) and “ScaleData”. The Batch effect between samples was corrected using Harmony v1.2.0.^[^ ^87^ ^]^ Then, the “FindNeighbors” with dims set to 1:30 and “FindClusters” with resolution of 0.6 were used to construct the Shared Nearest Neighbor (SNN) graph and to cluster the cells. Finally, “RunUMAP” was generated to visualize clusters. The cell type annotation of each cluster was combined by the following methods: 1) defined by known marker genes; 2) defined by significantly cluster‐enriched genes relative to the other clusters for all conditions. The marker genes of cell types and the top 100 genes highly expressed in each cell type are provided in Tables S5 and S6 (Supporting Information), respectively. The cluster‐enriched genes were computed by the “FindAllMarkers” function in Seurat v4.3.0^[^ ^84^ ^]^ with the following parameters: |log_2_FoldChange (FC)| greater than 0.25 and adjusted *P*‐value (p_adj) less than 0.05. Statistical significance was determined using the Wilcoxon rank‐sum test. The Spearman correlation coefficients among the percentages of cell types in two buffalo types were calculated with the ggpubr package (https://rpkgs.datanovia.com/ggpubr/) and visualized with the ggplot2 package v3.5.1.^[^ ^88^ ^]^

### Differential Expression Analysis and Gene Ontology Enrichment Analysis

The function of “FindMarkers” of Seurat v4.3.0^[^ ^84^ ^]^ was used to calculate differentially expressed genes (DEGs) between buffalo types based on a dual threshold of |log2 FC| > 0.25. The screening criteria for upregulated genes in river buffalo were p_adj < 0.05 and log2FC > 0.25, while for downregulated genes, the criteria were p_adj < 0.05 and log2FC < −0.25. DEGs in each sample were annotated using the biological process of GO terms based on the reference genome. GO enrichment analysis was performed using the clusterProfiler package v4.6.2^[^ ^89^ ^]^ and visualized with either the enrichplot v1.18.4^[^ ^90^ ^]^ or ggplot2 package v3.5.1.^[^ ^88^ ^]^

### Cell–Cell Interaction Analysis

To investigate cellular communication patterns between potential cell types, the CellChat package^[^ ^91^ ^]^ was used with default parameters, which was a manually curated database of literature‐supported ligand‐receptor interactions in humans and mice. To run CellChat analysis in buffalo datasets, buffalo gene symbols were mapped to human orthologs. Ligand‐receptor pairs with *P*‐value < 0.05 were considered to be significant.

### Principal Variance Component Analysis (PVCA)

In brief, to conduct the PVCA, the SoupX corrected raw counts for each gene and each biological sample were aggregated using the aggregateData function of the muscat package v.1.5.2.^[^ ^92^ ^]^ The resulting matrix was normalized by dividing each feature of a sample by the total counts from that sample, multiplying by 100,000, and scaling the result using the function log (x + 1). As variables, the sample annotation fields ‘Buffalo type’, ‘Tissue’, and ‘Cell lineage’ were considered. All variables (or combinations of such) not passing the threshold were summarized as ‘Other’ in the analysis. The residual was then defined as the remaining proportion of variance not being associated with any of the variables that are explanatory nor informative to a minor proportion. Then, PVCA was performed using the pvca package v1.46.0^[^ ^93^ ^]^ with the default parameters.

### Estimation of Buffalo Type and Tissue Contribution to Gene Expression Variation

The variancePartition package v1.28.9^[^ ^94^ ^]^ was used to estimate the proportion of variance explained for each gene by a joint model including buffalo type and tissue as explanatory variables. Briefly, this method fits a linear mixed model and estimates the proportion of variance explained by each explanatory variable. This framework was used to identify the main contributor for each gene.

### Measurement of *TRHDE* Expression and TRH Concentration

Based on cattle RNA‐seq data,^[^ ^95^, ^96^, ^97^ ^]^ five female dairy cattle and five female beef cattle were selected (Table S13, Supporting Information) to assess *TRHDE* expression in the pituitary gland. To determine TRH concentrations in serum, blood samples were collected from six female buffaloes‐three river and three swamp‐two months postpartum. Additionally, six first‐lactation female cattle were sampled, including three dairy cattle at two months postpartum and three beef cattle at four months postpartum. Blood samples were centrifuged 2 h after collection at 3500 rpm for 15 min. The serum was then collected and transferred into tubes and stored at −80 °C until analysis. TRH concentrations in serum were quantified using an Enzyme‐linked immunosorbent assay (ELISA) kit (Jianglai biology, JL35057) according to the manufacturer's instructions. All results were presented as the mean ± standard error of the mean (SEM).

### Enrichment Analysis Between Cell Types and Complex Traits

To uncover associations of traits with cell types, Fisher's exact tests were used to perform enrichment analyses for genes under selection in river buffalo (Table S7, Supporting Information)^[^ ^12^ ^]^ based on three gene sets, including cell‐type‐specific genes in tissues, DEGs between the two buffalo types, and genes highly expressed in river buffalo. Specifically, the genes were sorted in descending order by the FC for each cell type in each of the 12 tissues, and the top 700 genes were extracted from each of the three gene sets. Then, the 122 genes under selection in the river buffalo were collected.^[^ ^12^ ^]^ Finally, the significance level (*P*‐value) of the enrichment fold was calculated using Fisher's exact test with FDR correction, and a *P*‐value < 0.05 was defined as significant enrichment.

Meanwhile, the GWAS summary statistics on the autosomes of cattle for five milk production traits were collected, including SNP positions, p‐values, and marker effects.^[^ ^98^ ^]^ Genes in buffalo were mapped to their corresponding cattle orthologous genes using one‐to‐one mapping. The homologous genes were identified by OrthoFinder, a software that identifies orthologous genes by integrating the bidirectional best‐hit principle and analysis of phylogenetic trees of genes.^[^ ^99^ ^]^ The sum‐based marker‐set test approach^[^ ^77^ ^]^ was then applied, using 20‐kb windows around gene regions,^[^ ^100^, ^101^ ^]^ as implemented in the qgg package v1.1.2,^[^ ^102^ ^]^ to perform enrichment analysis. To obtain an empirical *P*‐value for two gene sets, including genes highly expressed in river and swamp buffalo, this permutation procedure was repeated 10,000 times and a one‐tailed test of the proportion of random summary statistics greater than that observed was employed. Based on scRNA‐seq data from human milk and breast tissue,^[^ ^68^ ^]^ the expression of intersected genes between upregulated genes in river buffalo and GWAS candidate genes in dairy cattle was visualized using the Nebulosa package v1.16.0.^[^ ^103^ ^]^

### Cross‐Species Single‐Cell Transcriptomic Analysis

For comparative analysis of the mammary gland between human and buffalo, SAMap v1.0.1247^[^ ^104^ ^]^ was employed to quantify cellular homology between the two species. Gene–gene relationship weights were computed based on BLAST bit scores derived from pairwise sequence alignments, followed by iterative clustering until alignment scores exceeded the default threshold for matched cell populations. Interspecies cellular correspondence was visualized as a heatmap, highlighting conserved cell‐type features between human and buffalo mammary tissues.

### Comparative Co‐Expression Network Analysis and Evolutionary Conservation in Buffalo and Human Mammary Tissues

Co‐expression network analysis on buffalo and human mammary tissues was performed using the Hotspot algorithm.^[^ ^105^ ^]^ A k‐nearest neighbor (k‐NN) graph was constructed using the create_knn_graph function with n_neighbors = 30, and only genes exhibiting statistically significant correlations (FDR < 0.05, Fisher's z‐test) were retained. Co‐expression modules were identified using the create_modules function with min_gene_threshold = 200 and fdr_threshold = 0.05. To assess module‐cell type associations, the top 200 marker genes per cell type were selected based on log2FC, and overlap significance was calculated using the odds ratio (OR) and Fisher's exact test, followed by FDR correction (Benjamini–Hochberg procedure). This approach enabled systematic identification of biologically relevant associations between co‐expression modules and cell‐type‐specific transcriptional programs.

To investigate the evolutionary conservation of co‐expression networks between buffalo and human mammary tissues, orthologous genes were identified using OrthoFinder.^[^ ^99^ ^]^ Preserved modules between species were computed using the function modulePreservation() with 1,000 permutations.^[^ ^106^ ^]^ Gene sub‐module preservation between networks was calculated using the R package GeneOverlap.^[^ ^107^ ^]^ The preservation significance was evaluated based on two key metrics: 1) the number of consistently co‐expressed orthologous genes, and 2) the statistical significance of module preservation assessed using Fisher's exact test with FDR correction. Modules showing significant conservation (FDR < 0.05) were considered to represent evolutionarily maintained co‐expression patterns.

### Ethics Statement

All experimental procedures in this study were approved by the Animal Welfare Committee of China Agricultural University (AW42303202‐2‐1).

## Conflict of Interest

The authors declare no conflict of interest.

## Author Contributions

D.D. and J.S. contributed equally to this study. Y.Z., L.F., and S.L.: conceptualization. D.D., J.S., L.J., B.H., X.W., S.Y., Y.Y., W.C., and H.M.: investigation. D.D., J.S., and K.W.: formal analysis, methodology and visualization. D.D.: validation. D.D., S.L., and K.W.: writing‐original draft. J.S., A.P., L.F., and Y.Z.: writing‐review and editing.

## Supporting information



Supporting Information

Supplemental Table 1

## Data Availability

Single‐cell RNA‐seq data have been deposited into the CNGB Sequence Archive of China National GeneBank DataBase (CNGBdb) with accession number CNP0006829. The scripts used are saved in GitHub (https://github.com/DongmeiDai/buffalo‐cell‐atlas).
