# Supplementary material for: Comparative Single‐Cell Transcriptomic Landscape Reveals the Regulatory Mechanisms of Lactation during Selective Breeding in Asian Water Buffalo
Source: Adv Sci (Weinh). 2025 Jul 11;12(37):e08847. doi: 10.1002/advs.202508847 (PMC12499429; doi:10.1002/advs.202508847)
Supplement: Supplementary file 1 — Supporting Information [file ADVS-12-e08847-s002.docx]

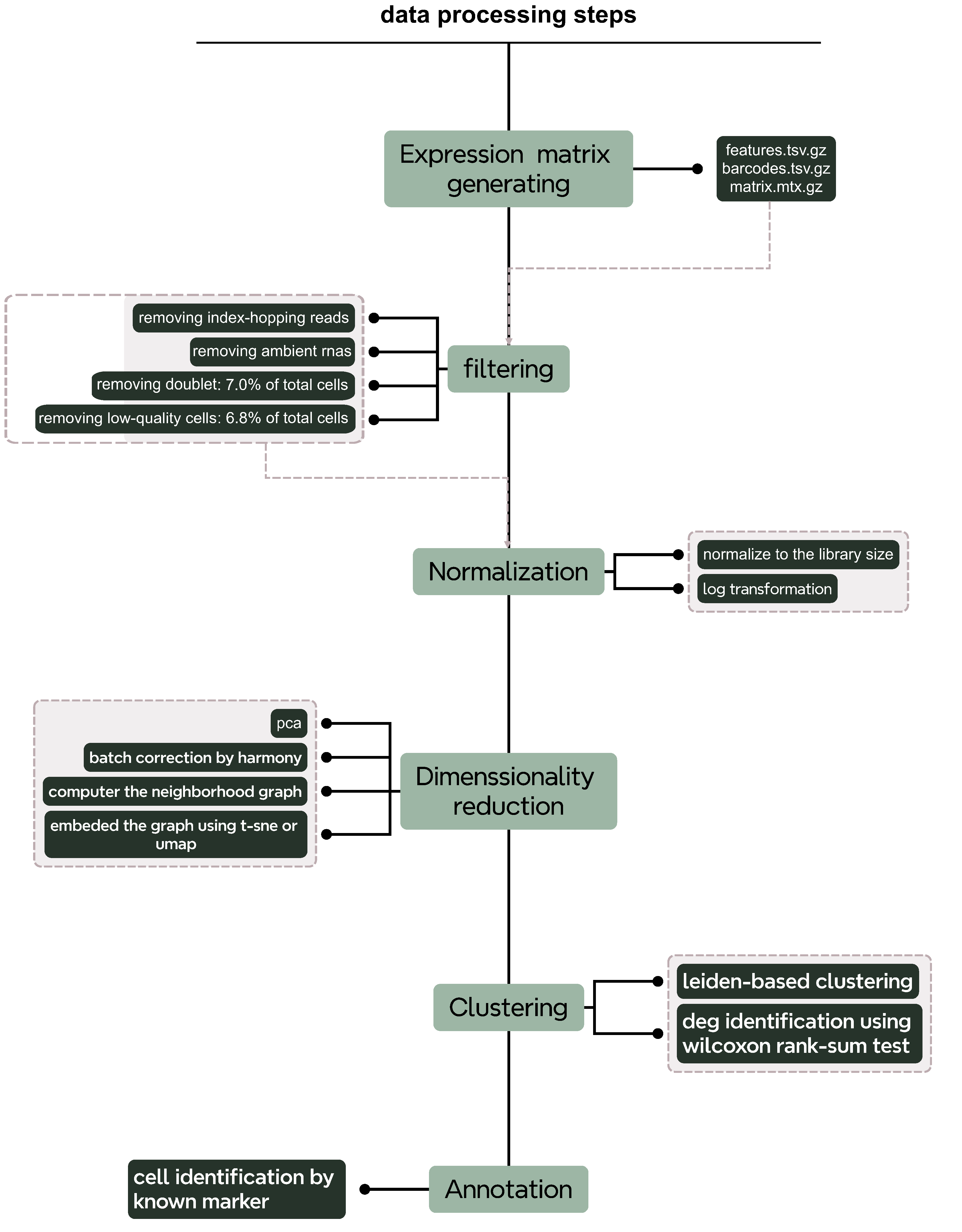


**Figure S1. The summary of data processing steps.**


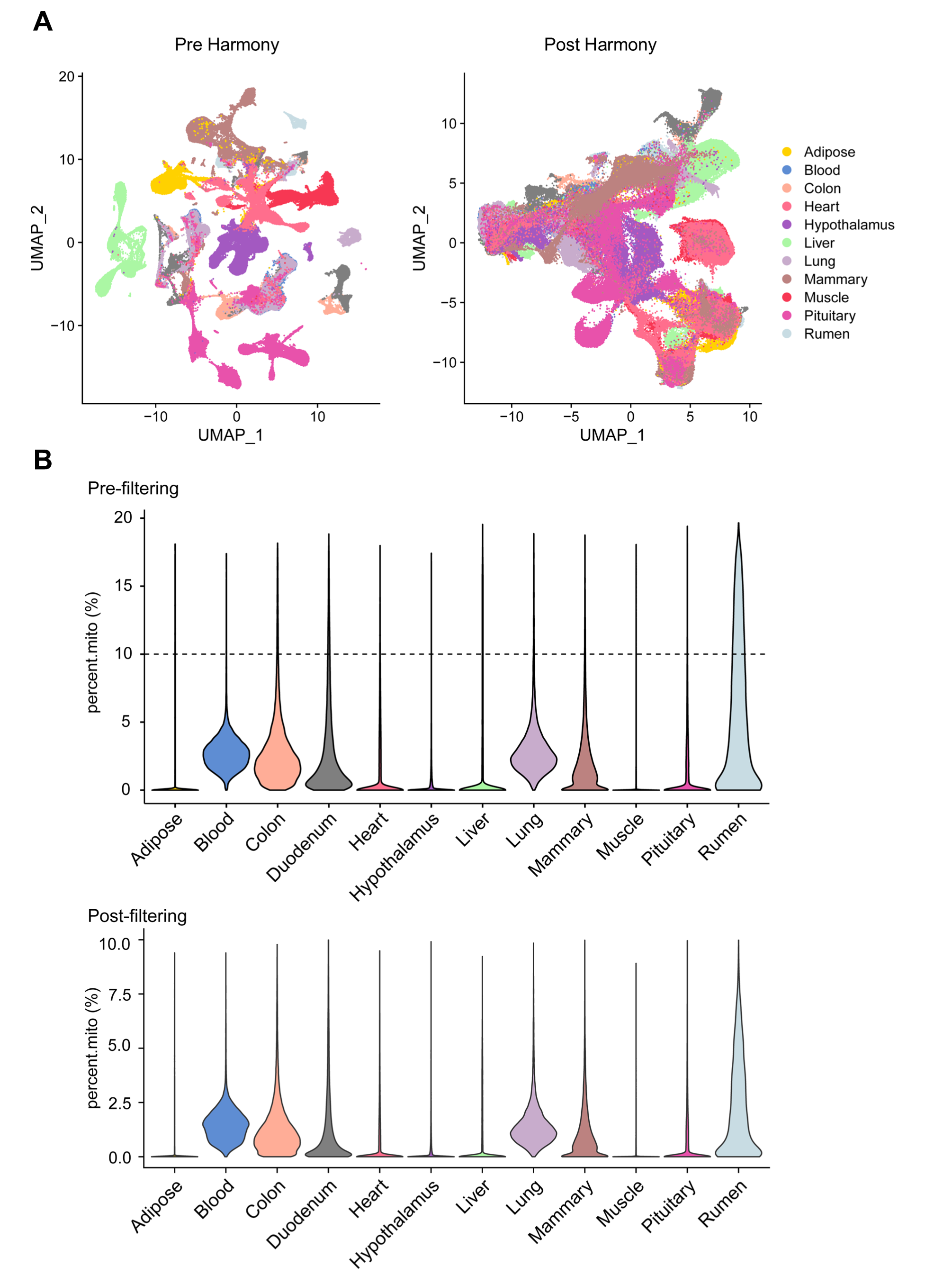
**Figure S2.** **Overview of the sc/snRNA-seq batch correction and quality control measures.** (A) UMAP visualization of cells colored by tissues before and after batch correction. (B) Violin plots showing the percentage of mitochondrial genes per cell across 12 tissues before and after quality control.


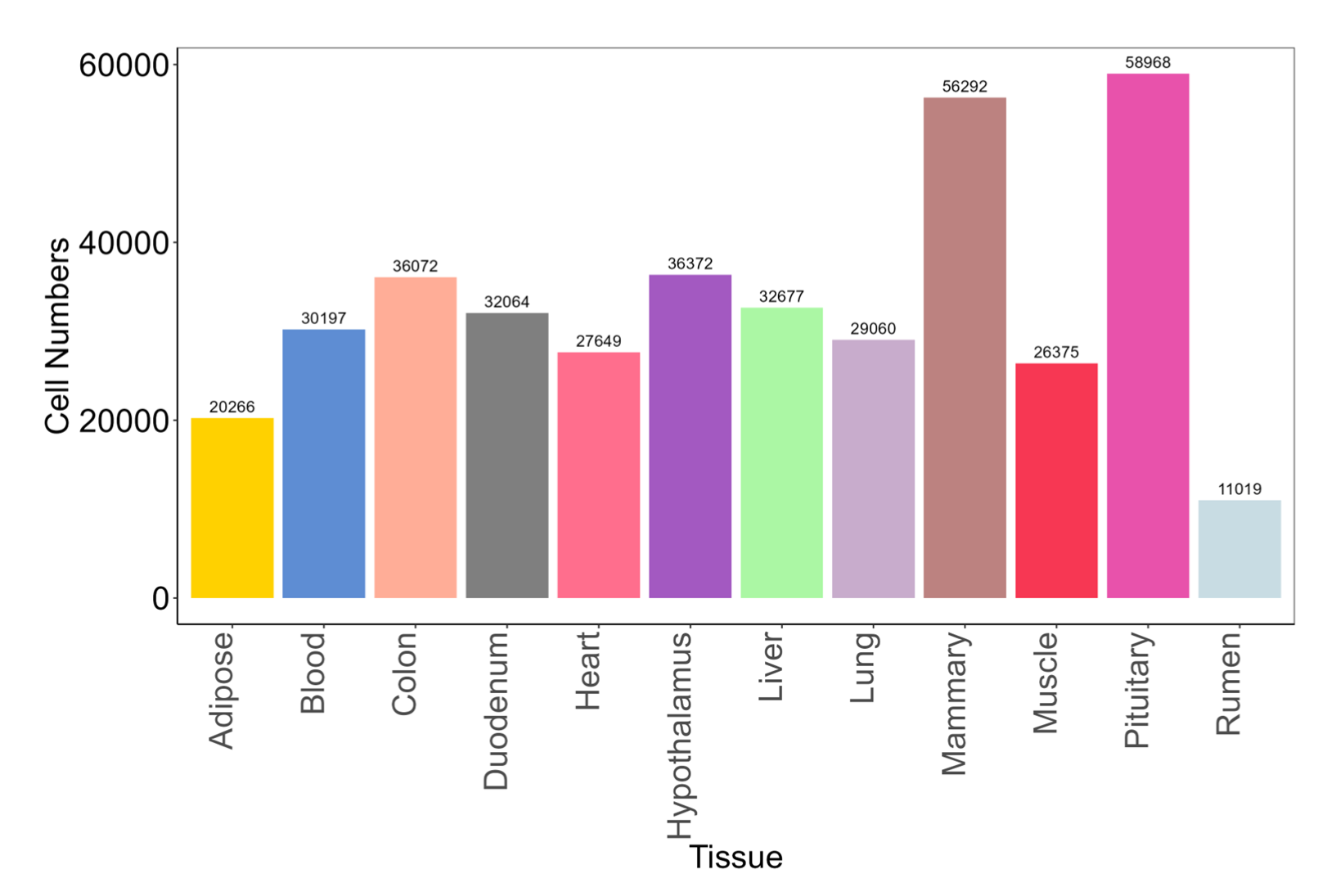


**Figure S3. Cell numbers in different tissues.**


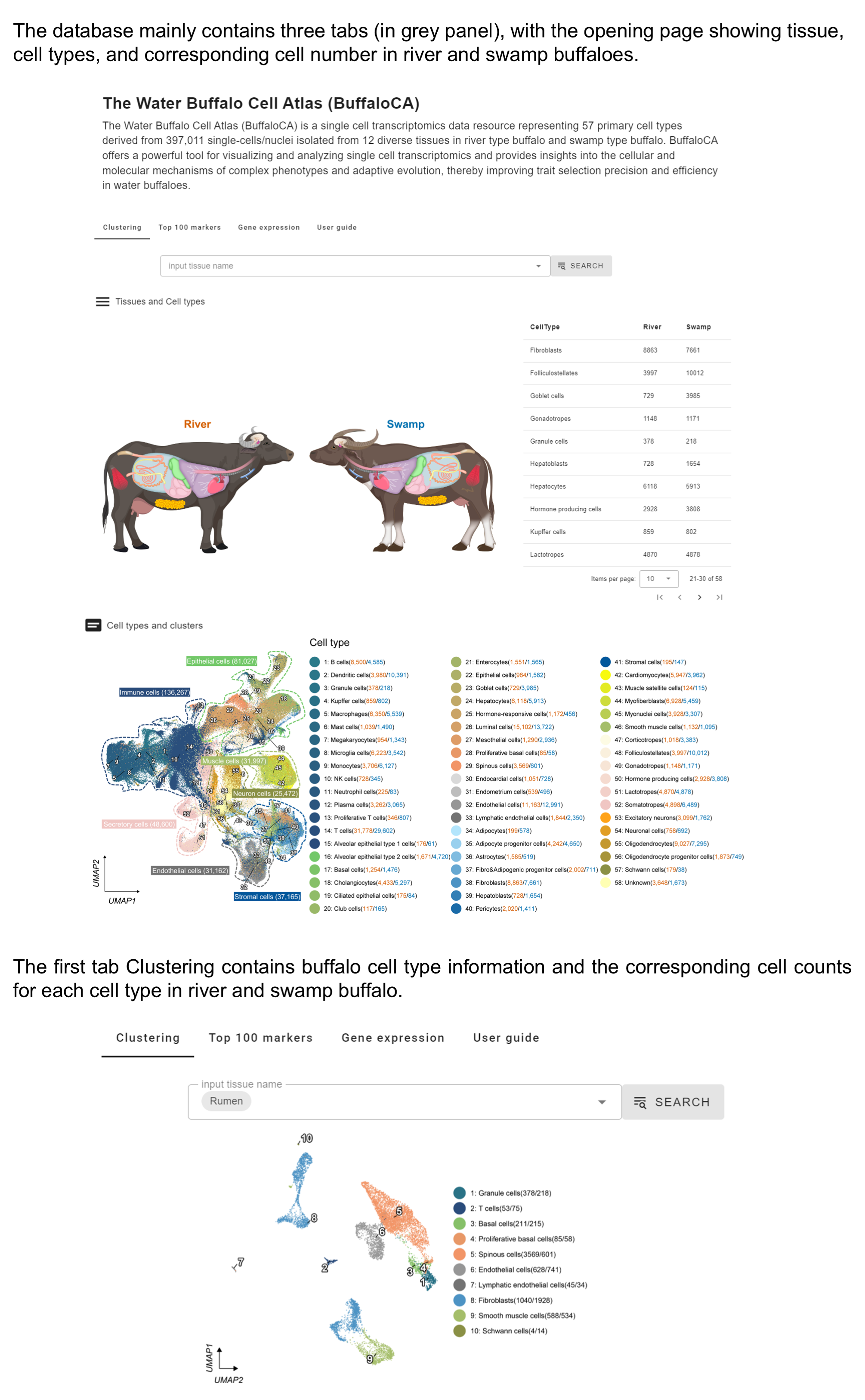


**Figure S4.** **Web portal screenshots showing the homepage and tissue clustering results.**


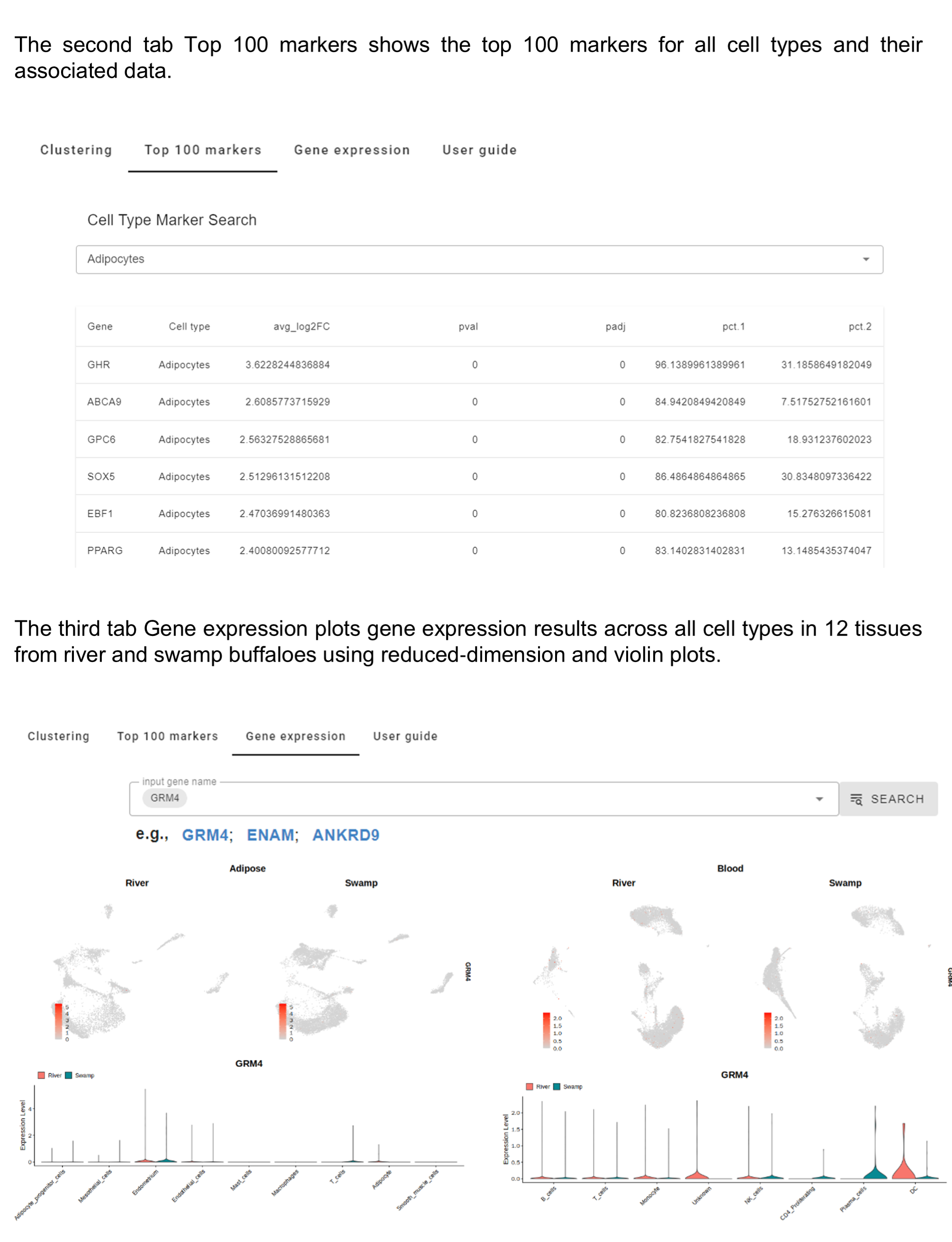


**Figure S5. Web portal interface displaying top 100 cell-type marker genes and gene expression exploration tools.**


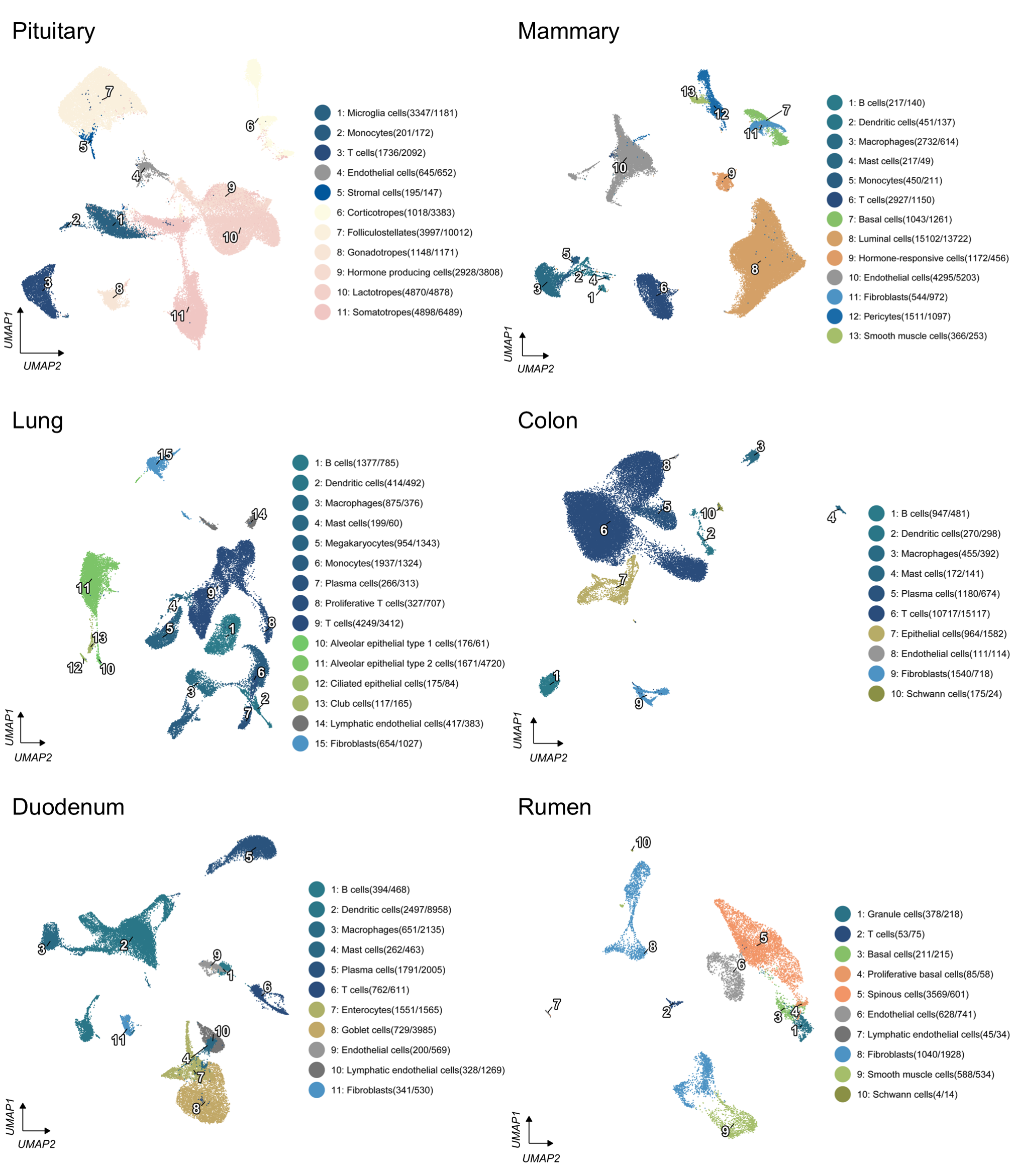
**Figure S6. Buffalo cell cluster annotations.** UMAP visualization of cell clusters in the pituitary, mammary, lung, colon, duodenum, and rumen. The left number in parentheses indicates the number of cells for river buffalo, and the right number for swamp buffalo.


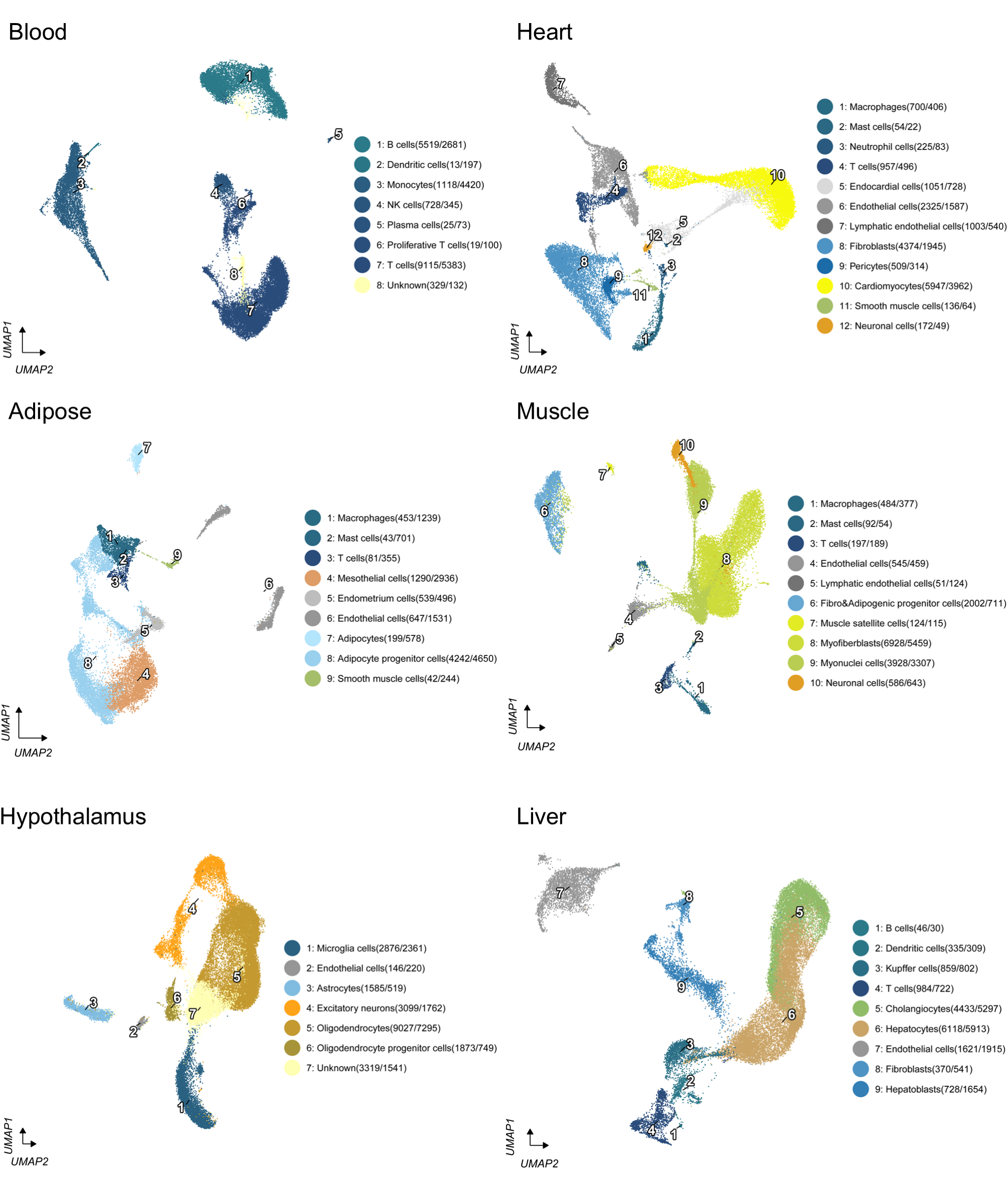
**Figure S7. Buffalo cell cluster annotations.** UMAP visualization of cell clusters in the blood, heart, adipose, muscle, hypothalamus, and liver. The left number in parentheses indicates the number of cells for river buffalo, and the right number for swamp buffalo.


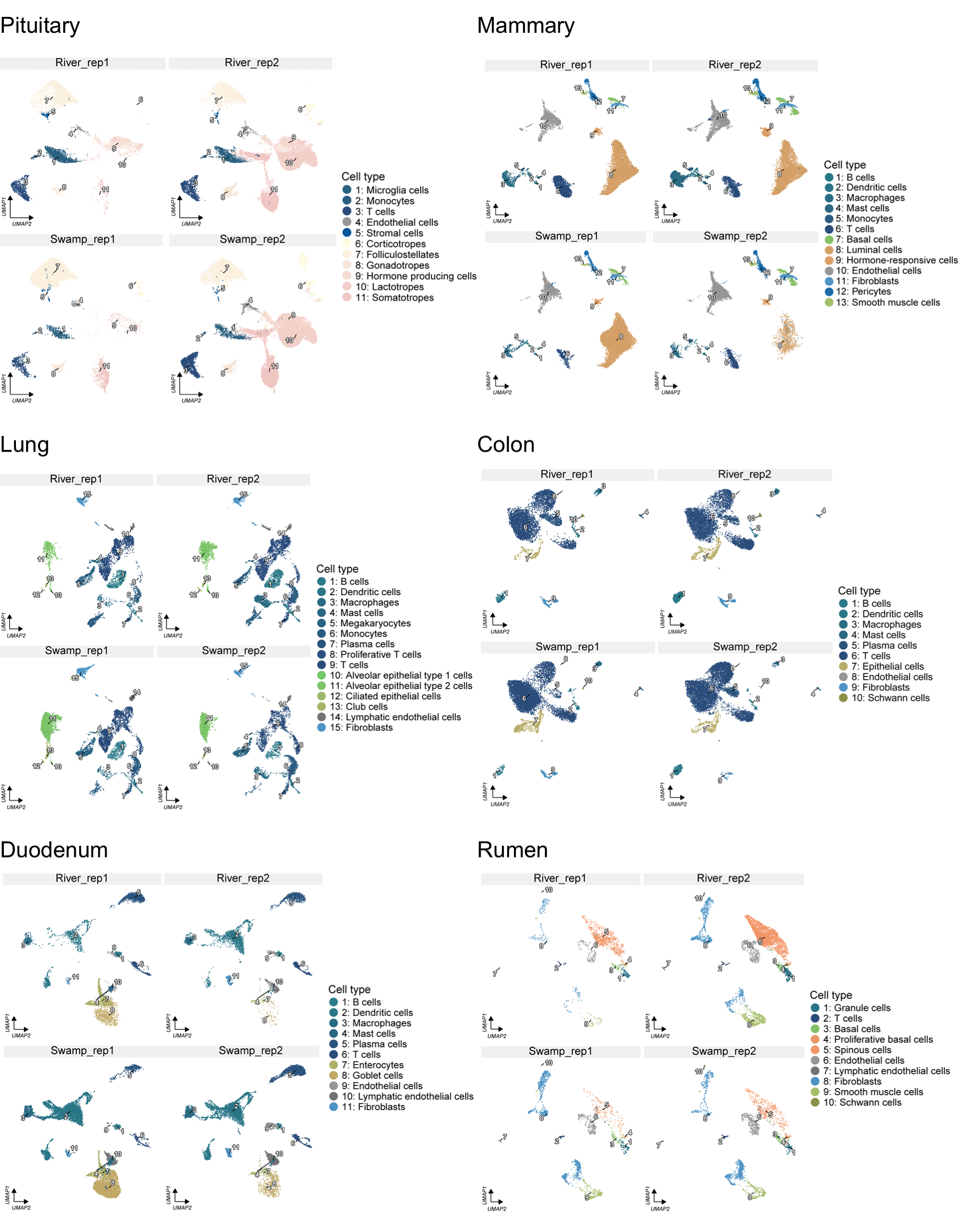


**Figure S8. UMAP visualization of cell types in the pituitary, mammary, lung, colon, duodenum, and rumen of four buffaloes.**


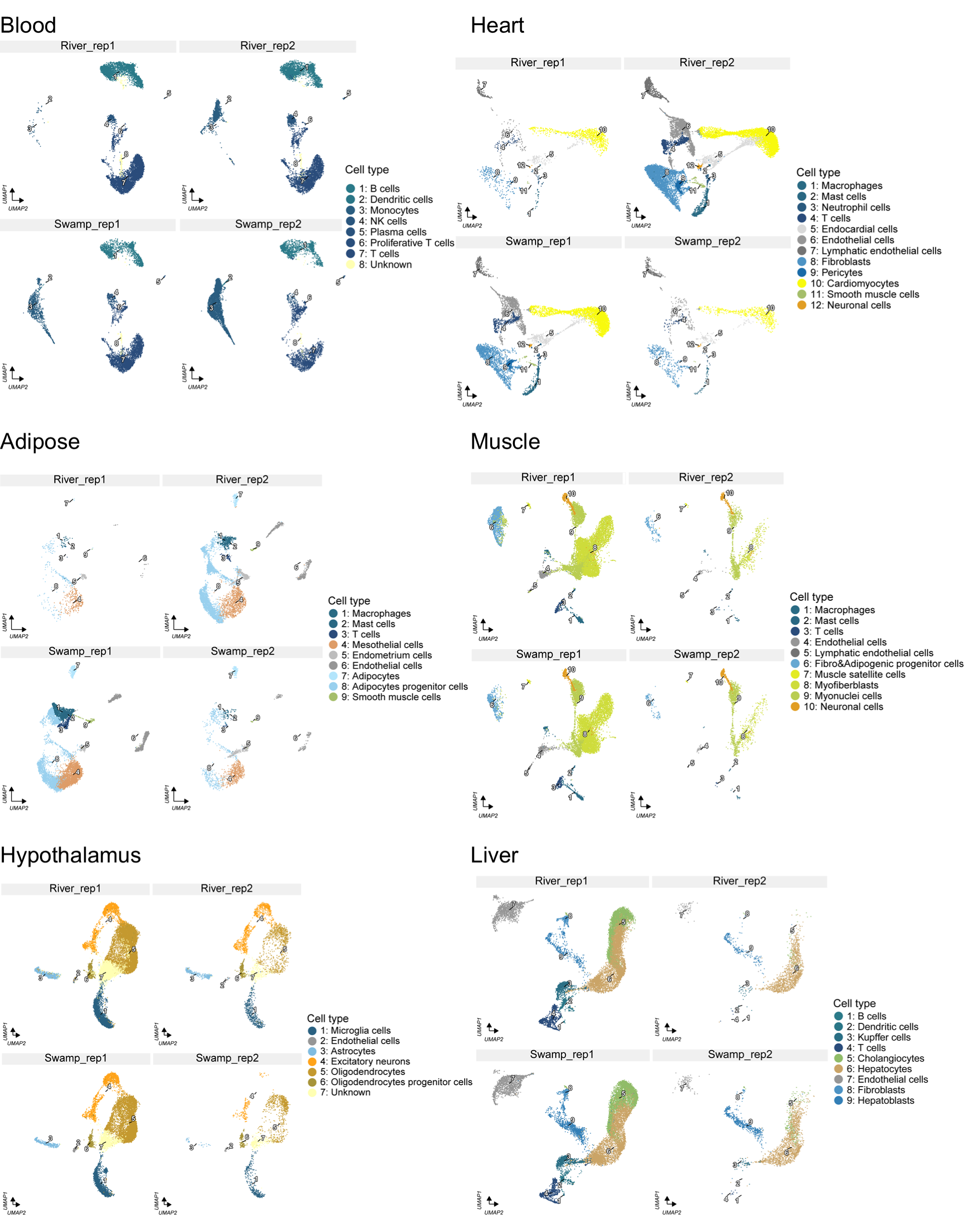


**Figure S9. UMAP visualization of cell types in the blood, heart, adipose, muscle, hypothalamus, and liver of four buffaloes.**


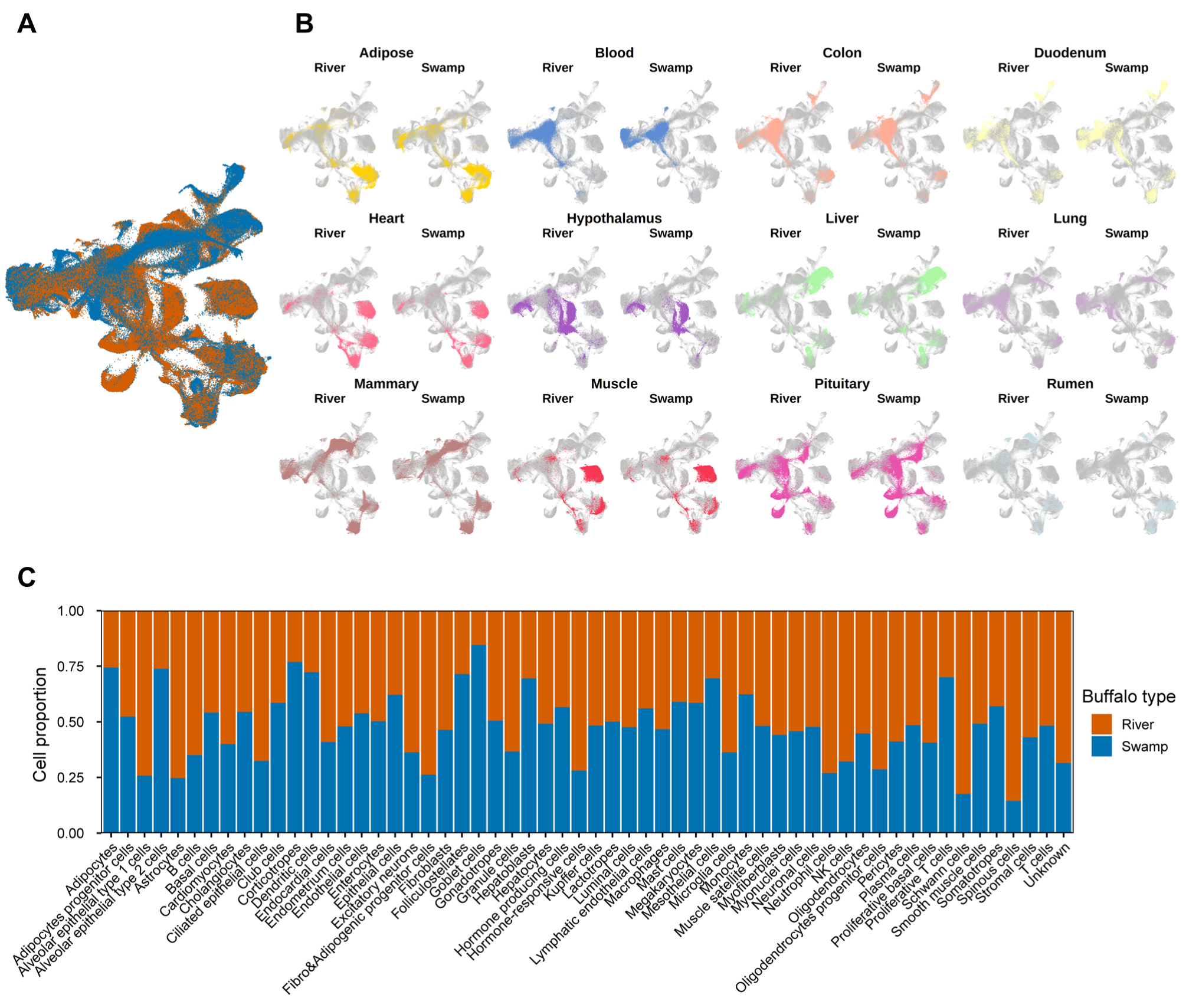


**Figure S10. Cell-type composition across tissues in river and swamp buffalo.** (A) UMAP visualization of all clusters, colored by buffalo types (orange: river buffalo; blue: swamp buffalo). (B) UMAP visualization of clusters stratified by tissue origin (12 tissues), colored by tissue types. (C) Proportional distribution of cell types across buffalo types, colored by buffalo types.


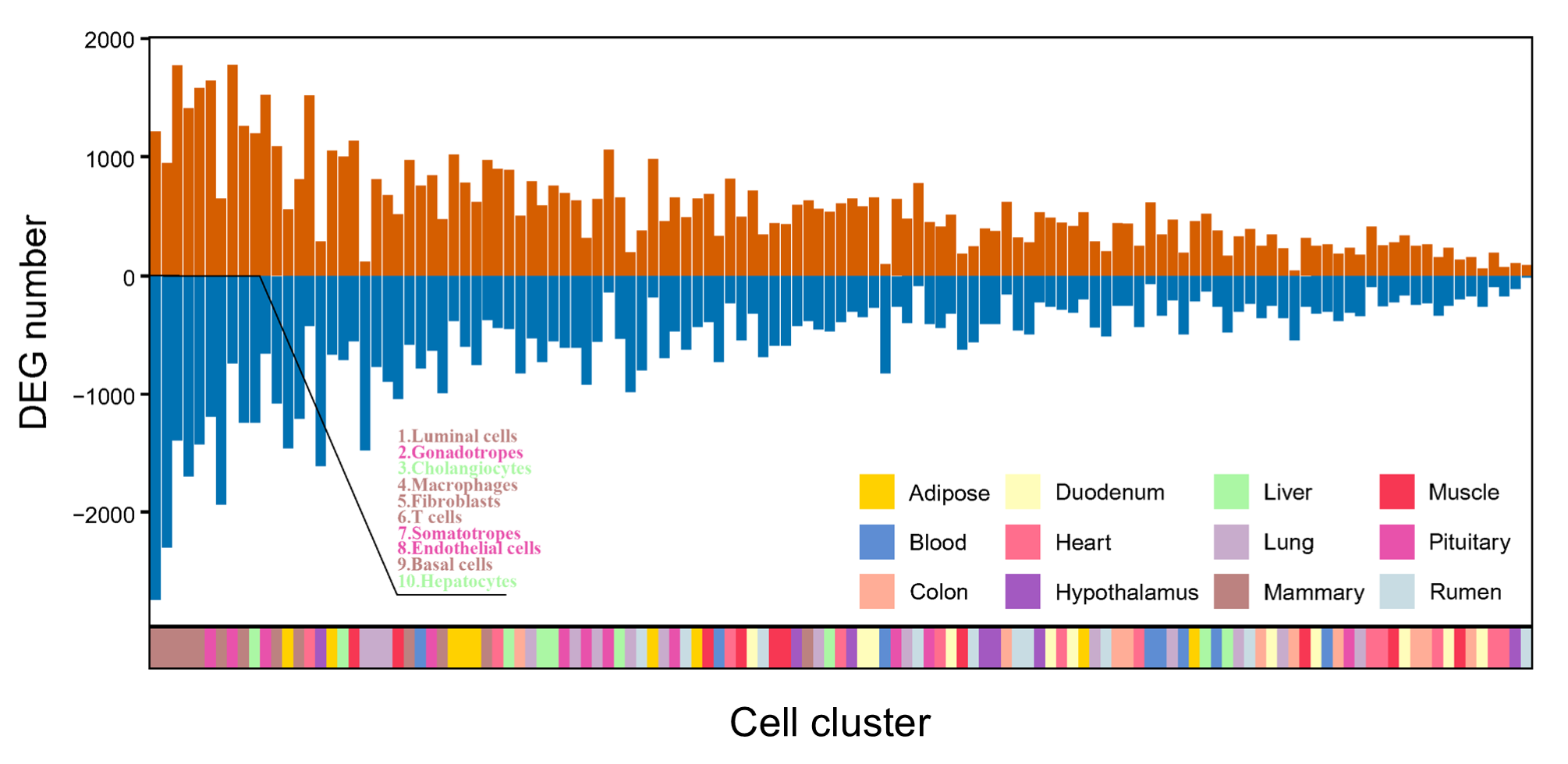


**Figure S11.** **The number of differentially expressed genes (DEGs) in each cell type across 12 tissues.** Orange represents genes highly expressed in river buffalo, and blue represents genes highly expressed in swamp buffalo.


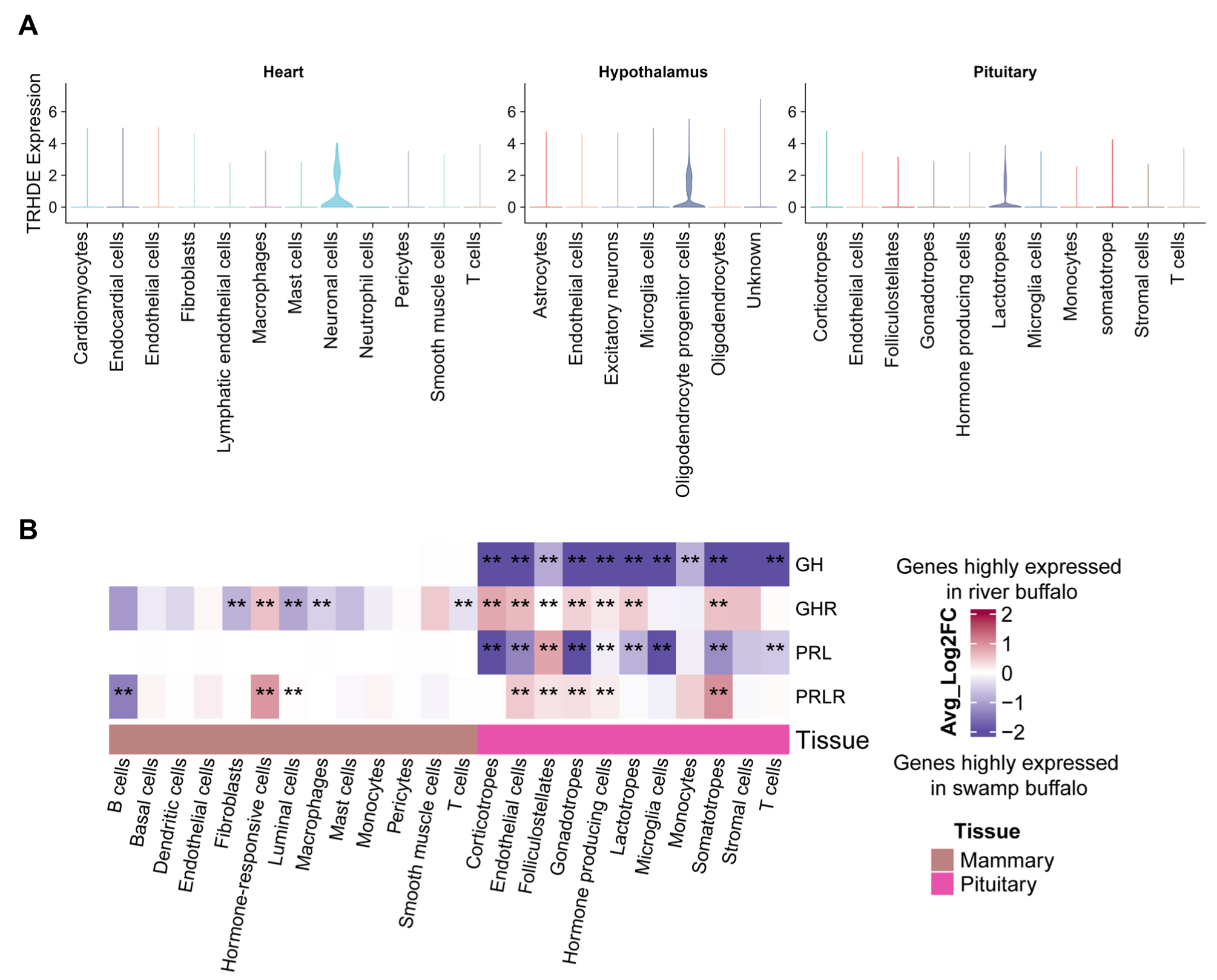


**Figure S12. Expression of lactation-related genes in river and swamp buffalo.** (A) *TRHDE* expression across all cell types. (B) Heat map showing the expression differences of *PRL*, *GH* and their receptor genes (*PRLR* and *GHR*) in mammary and pituitary cell types of river and swamp buffalo.


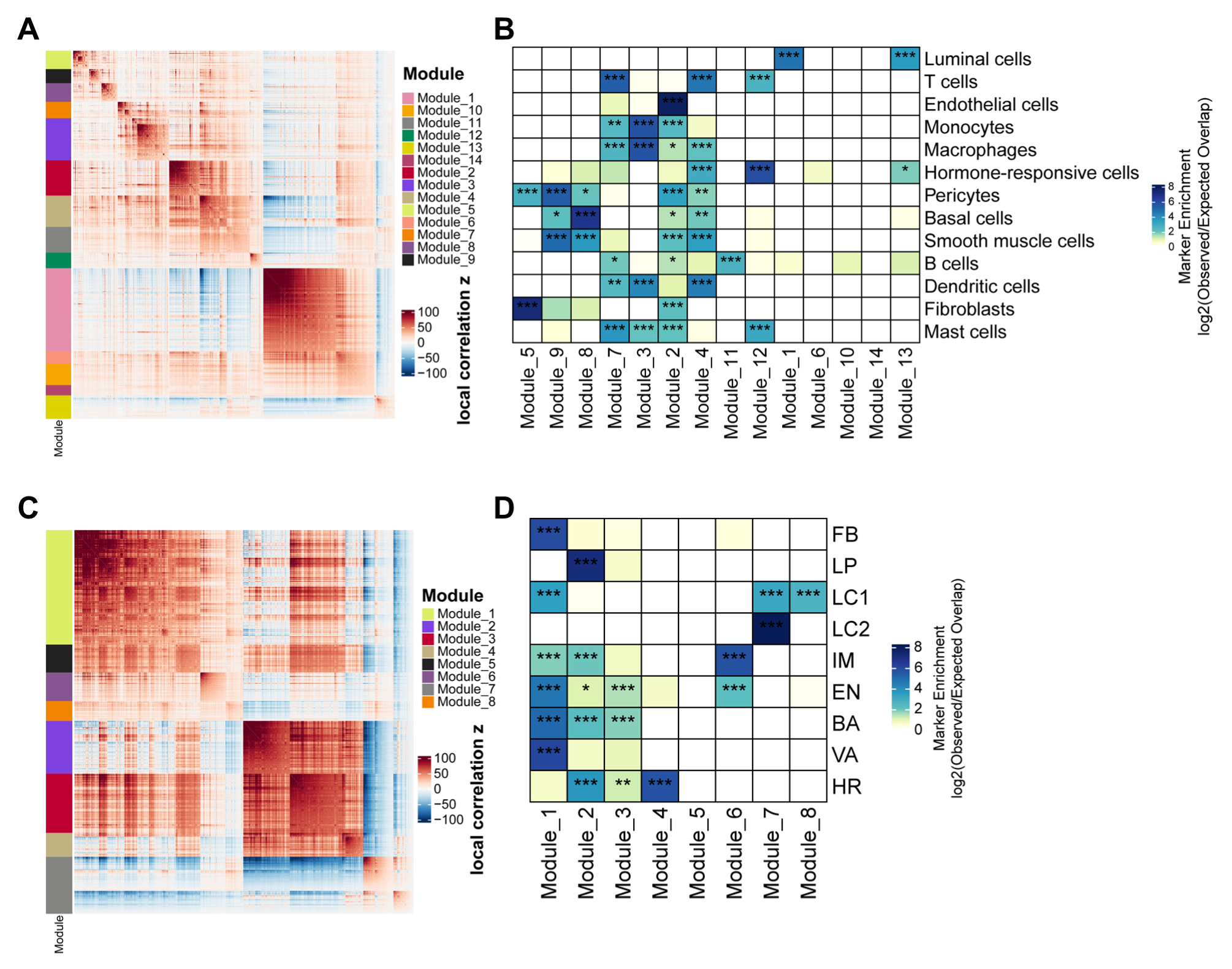


**Figure S13. Co-expression network analysis in buffalo and human mammary tissues.** (A) Hotspot modules in buffalo mammary cells. The heatmap displays 7,311 genes with significant autocorrelation (FDR < 0.05), grouped into 14 distinct co-expression modules in buffalo mammary gland. (B) Correlation analysis between gene co-expression modules and cell types in buffalo mammary gland. Statistical significance is denoted as follows: * denotes p_adj < 0.05, ** denotes p_adj < 0.01, *** denotes p_adj < 0.001. (C) Hotspot modules in all human milk and mammary cells. The heatmap displays 4,949 genes with significant autocorrelation (FDR < 0.05), grouped into eight distinct co-expression modules in human milk and mammary gland. (D) Correlation analysis between gene co-expression modules and cell types in human milk and mammary gland. Statistical significance is denoted as follows: * denotes p_adj < 0.05, ** denotes p_adj < 0.01, *** denotes p_adj < 0.001.
